# A novel extracytoplasmic function sigma factor (RpoE6) regulates biogenesis of a minor flagellin and other functions in *Azospirillum brasilense* Sp7

**DOI:** 10.1128/jb.00464-25

**Published:** 2025-12-29

**Authors:** Parikshit Gupta, Hahley Wiltse, Parul Pandey, Sushant Rai, Vijay Shankar Singh, Sambuddha Talukdar, Gladys Alexandre, Anil Kumar Tripathi

**Affiliations:** 1 School of Biotechnology, Banaras Hindu University, Institute of Science 30114, Varanasi, India; 2 Biochemistry and Cellular and Molecular Biology Department, University of Tennessee, Knoxville, Tennessee, USA; 3 Department of Microbiology, School of Life Sciences, Sikkim University 179229, Gangtok, Sikkim, India; 4 Department of Biological Sciences, Indian Institute of Science Education and Research, Mohali (IISER Mohali), Mohali, Punjab, India; University of Massachusetts, Chan Medical School, Worcester, Massachusetts, USA

**Keywords:** *Azospirillum brasilense*, swimming motility, extracytoplasmic function sigma factor, small flagellin, PQQ

## Abstract

In Gammaproteobacteria, the biogenesis of the flagellar filaments is regulated by a flagellum-specific sigma factor (FliA). In Alphaproteobacteria, however, a flagellum-specific sigma factor is not known. In our search for a FliA-like sigma factor in *Azospirillum brasilense* Sp7, we found an extracytoplasmic function sigma factor (RpoE6) located in a gene cluster consisting of FlgK, FlgL, FliD, FliS, and FliC2. A strain lacking RpoE6 (Δ*rpoE6*) was compromised in expressing a small flagellin, FliC2, which contributes to the polar flagellum structure and function in swimming motility, though there was no detectable defect in swimming motility in swim plates. A non-polar inactivation of this sigma factor also led to a growth defect that depended on aeration. FliC2, like the major flagellin of the polar flagellum, is also likely glycosylated. A hypothetical protein, possibly behaving as an anti-sigma factor (Asf6), encoded downstream of *rpoE6*, interacts with RpoE6. A mutant lacking Asf6 (Δ*asf6*) overproduces FliC2 and possesses thinner flagellar filaments compared to the wild type, suggesting that the abundance of FliC2 is oppositely regulated by RpoE6 and Asf6. Overexpression of *rpoE6* in *A. brasilense* upregulates many proteins, including those involved in flagellar filament (FliD, FliS, and FliC2), required for growth on fructose and glycerol, as well as in the upregulation of another sigma factor, RpoE8. Our data support a role for RpoE6 in coupling FliC2 expression with genes involved in glycerol and fructose metabolism. Conservation of the genomic organization of *rpoE6* in related Alphaproteobacteria suggests a similar function in other species.

**IMPORTANCE:**

*Azospirillum brasilense* is a common plant growth-promoting rhizobacterium that colonizes crop rhizospheres using chemotaxis via its polar flagellum. However, our understanding of the structure, composition, and biogenesis of the polar flagellum in *A. brasilense* is limited. Unlike Gammaproteobacteria, where FliA and FlgM regulate flagellum biogenesis, no sigma or anti-sigma factors are known to regulate chemotactic motility in Alphaproteobacteria, including *A. brasilense*. This study identifies an extracytoplasmic function sigma factor (RpoE6) and its anti-sigma factor (Asf6) which modulate the expression of the minor flagellin FliC2, thereby altering the polar flagellum structure. Unlike FliA and FlgM, this regulatory pair also controls other functions such as glycerol and fructose utilization. To our knowledge, this is the first report of an alternative sigma factor regulating a flagellin in Alphaproteobacteria.

## INTRODUCTION

Bacteria of the genus *Azospirillum* are plant growth-promoting rhizobacteria that colonize the roots of a wide variety of dicot and monocot plants. These bacteria promote growth through phytohormone production and nitrogen fixation (1). *Azospirillum brasilense* is one of the most often isolated bacterial species from plant rhizospheres and is frequently used as a biofertilizer for plant growth promotion. Motility and chemotaxis are two important phenotypic traits that are essential for plant root colonization (2–5). *A. brasilense* possesses two types of flagella: the polar flagellum is required for swimming through a liquid medium, and multiple lateral flagella are induced upon swarming in a viscous medium or over surfaces (6–8). The polar flagellum is also thought to mediate the adsorption of *A. brasilense* cells to the roots of wheat plants (9) and contribute to biofilm formation (10).

Our knowledge about the structure, function, and regulation of bacterial flagella is largely based on Gammaproteobacteria, including *Escherichia coli*, *Salmonella typhimurium*, *Vibrio cholerae*, *Pseudomonas aeruginosa*, *Helicobacter pylori,* etc. A typical flagellum consists of the basal body, the hook (FlgE), the hook-filament junction (FlgK, FlgL), the filament (FliC), and the filament cap (FliD). The basal body is embedded in the cell envelope, while the hook (FlgE) and filament (FliC) extend to the exterior of the cell body. The junction (FlgK and FlgL) connects the filament to the hook. A type III protein export apparatus is located at the flagellar base and transports flagellar proteins from the cytoplasm to the distal end of the growing flagellar structure. During assembly of the basal body, the export apparatus transports rod (FliE, FlgB, FlgC, FlgF, FlgG, and FlgJ) and hook (FlgD, FlgE, and FliK) proteins. Upon completion of the basal body, FlgE polymerizes into the hook structure with the help of the hook cap FlgD. When the hook reaches its mature length, the type III protein export apparatus switches its export specificity (11), thereby terminating the export of hook proteins (FlgD, FlgE, and FliK) and initiating the export of filament proteins (FlgK, FlgL, FliD, and FliC) (12, 13). Then, the hook cap is replaced by self-assembling FlgK, which, together with FlgL, forms the hook-filament junction in this order. The filament cap (FliD) is located at the tip of the growing filament and promotes filament formation by assembling flagellin (FliC) molecules (14–16). FlgN, FliT, and FliS act as flagellar type III export chaperones, with flgN being specific for FlgK and FlgL, and FliT and FliS for FliD and FliC, respectively.

In *Escherichia coli,* the flagellar regulon consists of more than 60 genes that are organized in three hierarchically ordered transcriptional classes, Classes 1, 2, and 3, which are temporally regulated upon induction. The early or Class 1 genes comprise the *flhDC* operon encoding the master regulator of the entire flagellar cascade, FlhD_4_C_2_ (17), which controls the expression of Class 2 genes. The middle or Class 2 genes encode components of the flagellar basal body and a flagellum-specific sigma factor, FliA, which regulates the expression of late or Class 3 genes, encoding proteins that make the flagellar filament (FlgK, FlgL, FlgM, FliD, and FliC) and control motility and chemotaxis (18). Prior to the completion of the basal body, an anti-sigma factor (FlgM) binds to FliA and suppresses transcription of the Class 3 genes, including *fliC* (19, 20). The flagellar hook basal body (HBB) forms a type III secretion system capable of secreting both ﬂagellar sub-units and FlgM. The release of FlgM leads to the activation of FliA, which initiates the expression of Class 3 genes needed after HBB completion (21, 22). The Class 3 operons are controlled positively by FliA and negatively by FlgM. It has been shown that FlgM is an anti-sigma factor, which binds FliA to prevent its association with RNA polymerase core enzyme (21, 22). Therefore, the FliA-FlgM regulatory system has been believed to control specifically the formation of the flagellar filament.

Extracytoplasmic function (ECF) sigma factors are necessary for bacteria to sense and respond to extracellular stresses, but some ECF sigma factors respond to intracellular stress signals as well (23). Although *E. coli* harbors only one ECF sigma factor, the genomes of Alphaproteobacteria encode multiple ECF sigma factors (24). *A. brasilense* has been previously shown to have 10 ECF sigma factors (25). RpoE1, RpoE2, RpoE7, and RpoE10 have been previously characterized (25–28). Each ECF sigma factor has a specific regulon and is activated under a specific stress (29). Another hallmark of a typical ECF sigma factor is the presence of an anti-sigma factor that is encoded close to the sigma factor gene in the genome (30). These genes are commonly co-transcribed, and the canonical anti-sigma factor commonly regulates the activity of the sigma factor through protein-protein interactions. However, RpoE10 is an exception and does not have a canonical anti-sigma factor; it instead has an extended region within the protein that regulates the sigma factor activity, similar to an anti-sigma factor.

Despite the importance of flagellar motility in root colonization by *A. brasilense,* current knowledge about the composition, biogenesis, and regulation of the polar and lateral flagella in *A. brasilense* Sp7 and other Alphaproteobacteria is limited. The polar flagellum in *A. brasilense* Sp7 is reported to consist of a flagellin with a predicted size of 65 kDa, but it migrates at 100 kDa during SDS-PAGE due to glycosylation (31). The σ^54^ (RpoN) is required for the motility of *A. brasilense*, as a *rpoN::km* mutant of *A. brasilense* Sp7 is non-motile due to the lack of both lateral and polar flagella (32). The lateral flagella in *A. brasilense* Sp7 consist of the Laf1 flagellin of about 45 kDa (25). Inactivation of the gene encoding Laf1 in *A. brasilense* Sp7 produces a *laf1:km* mutant, which lacks lateral flagella but still produces a polar flagellum (33) and is able to swim (polar flagellum-dependent) but not swarm (lateral flagella-dependent). The biosynthesis of lateral flagella is closely related to *A. baldaniorum* Sp245 and is regulated by an ECF41-type sigma factor, RpoE10 (25). RpoN and RpoE10 are known to regulate the biosynthesis of flagellar proteins, and no homologs of FliA or FlgM were found in the genomes of *A. brasilense* or *A. baldaniorum*.

The aim of this study was to examine whether RpoE6 of *A. brasilense* Sp7, like FliA of *E. coli*, is involved in regulating the expression of its neighboring genes (*fliD*, *fliS*, and *fliC*), which are known to be Class 3 type genes involved in the biogenesis of the flagellar filament. Since the flagellin encoded in the vicinity of *rpoE6* is unusually small (28 kDa), and the role of such flagellins is not known in Alphaproteobacteria, we also investigated the importance of this flagellin for the swimming motility of *A. brasilense* Sp7. In addition, we found that RpoE6 regulates not only the expression of flagellar proteins, but it also regulates other functions, such as glycerol and fructose metabolism. Together, the data obtained here suggest that RpoE6 represents a previously uncharacterized mode of regulation of a polar flagellin, which affects the polar flagellum structure.

## RESULTS

### Genomic organization of RpoE6

**Fig 1:**
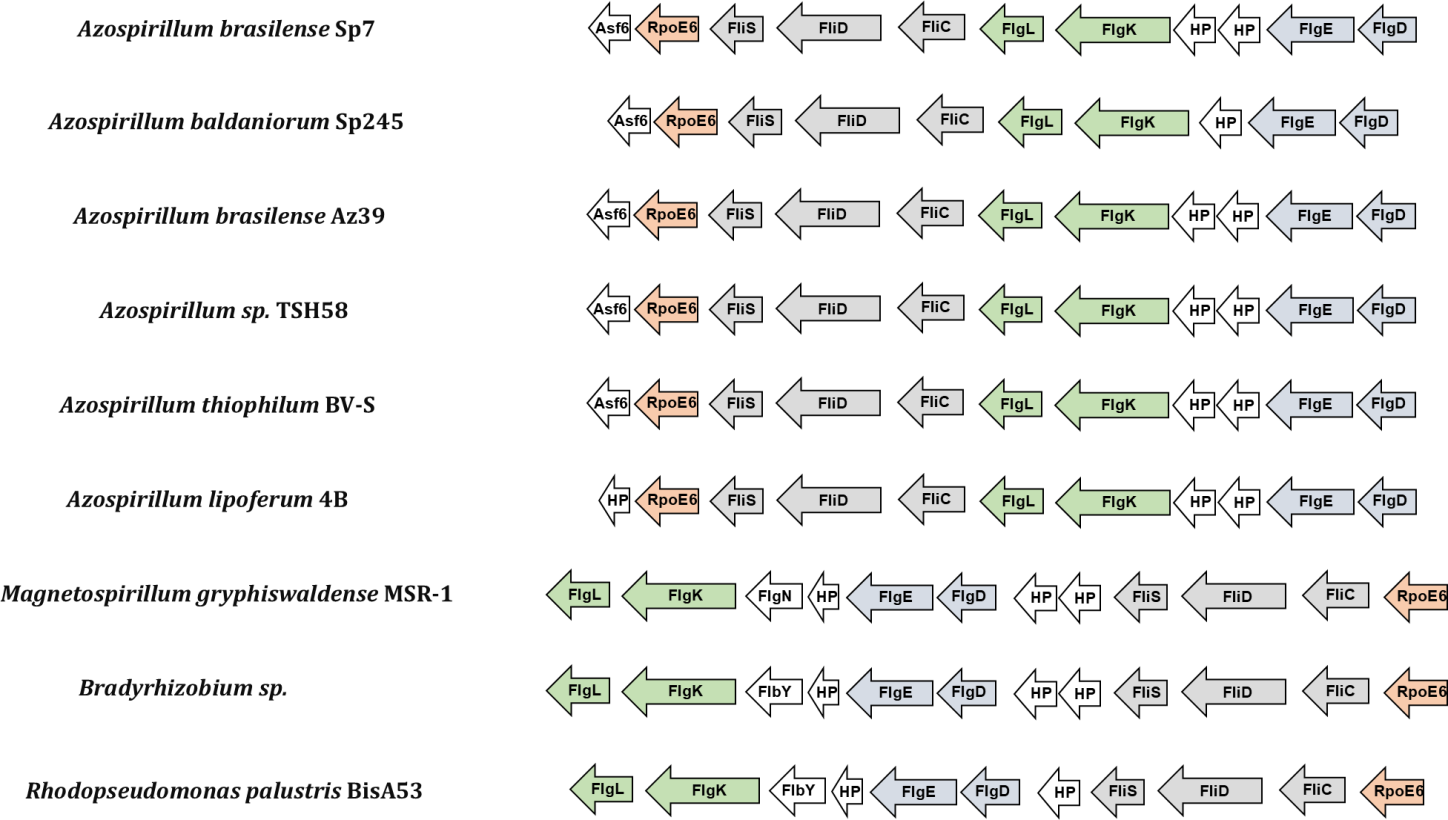
Synteny of the gene cluster encoding RpoE6, Asf6, and flagellar proteins (FlgD, FlgE, FlgK, FlgL, FliC, FliD, and FliS) in *Azospirillum brasilense* Sp7, *Azospirillum baldaniorum* Sp245, *Azospirillum brasilense* Az39, *Azospirillum* sp. TSH58, *Azospirillum thiophilum* BV-S, *Azospirillum lipoferum* 4B, *Magnetospirillum gryphiswaldense* MSR-1, *Bradyrhizobium* sp., and *Rhodopseudomonas palustris* BisA53. The pointed arrows indicate the direction of the genes.

RpoE6 (NCBI Locus: WP_035682069; Gene Locus Tag: OH82_RS01115; a homolog of AZOBR_p340058) is an ECF sigma factor of 192 aa. The gene encoding RpoE6 in *A. brasilense* Sp7 and other species of *Azospirillum* is the penultimate (9th) gene of a gene cluster comprised of 10 genes: *flgD*, *flgE*, *hp2*, *flgK*, *flgL*, *fliC*, *fliD*, *fliS*, *rpoE6,* and *hp1* (*asf6*) (Fig. 1). This gene cluster was missing in the annotated genome of *A. brasilense* Sp7 but was found in the genome sequence of *A. brasilense* Sp7 submitted by us in the GenBank (assembly: GCA_008274945.1) and other species of *Azospirillum* including *A. baldaniorum*. Comparison of this gene cluster with those present in other species of the genus *Azospirillum* showed a very high level of synteny, including in *A. lipoferum*, *A. thiophilum*, *A. baldaniorum*, *Azospirillum* Az39, and *Azospirillum* TSH58. It also showed notable synteny with the gene clusters of *Rhodopseudomonas palustris* BisA53, *Bradyrhizobium japonicum,* and *Magnetospirillum gryphiswaldense* MSR-1, except that the *rpoE6* homologs in these bacteria are the first genes of the cluster instead of being the last. In these bacteria, the *rpoE6* ortholog (*sigW*) is followed by *fliC* (NCBI Locus WP_035682079, Gene Locus Tag: OH82_RS01130), *fliD* (OH82_RS01125), *fliS* (OH82_RS01120), *hp*, *hp*, *flgD*, *flgE*, *hp*, *flgN*, *flgK,* and *flgL*. An alignment of the deduced amino acid sequence of RpoE6 of *A. brasilense* Sp7 shows more than 50% identity with its orthologs in *R. palustris* BisA53 (RPE_0783), and *B. japonicum* (BRADO4891), with a maximum of 54% identity with that of *M. gryphiswaldense* MSR-1: (MSR1_01160) (Fig. S1).

### Overexpression of RpoE6 upregulates flagellar proteins FliC2, FliS, and FliD

To decipher the role of RpoE6 in *A. brasilense* Sp7, we identified differentially expressed proteins caused by RpoE6 overexpression. To do this, we compared the proteomes of wild-type (WT) *A. brasilense* Sp7 and its derivative expressing RpoE6 via an IPTG-inducible promoter by high-resolution accurate mass spectrometer. There were 86 proteins that were detected only in *A. brasilense* Sp7 overexpressing RpoE6 and 100 proteins that were upregulated by at least twofold in *A. brasilense* Sp7 overexpressing RpoE6. Of these, FliD, FliS, FlgL, and FliE were detected only in *A. brasilense* Sp7 overexpressing RpoE6, whereas the previously uncharacterized small polar flagellin (named here FliC2) was upregulated by 64-fold (Table 1). In addition, we noted the upregulation of an operon consisting of RpoE8 (AMK58_24940), a hypothetical protein (AMK58_24945), and a periplasmic heavy metal sensor (AMK58_24950). Furthermore, we noted the upregulation of a gene cluster consisting of a Von Willebrand type A (VWA) domain-containing protein (AMK58_03235), a Sel1 repeat family protein (AMK58_03240), a putative virulence factor (AMK58_03245), a hypothetical protein (AMK58_03250), a hypothetical protein (AMK58_03255), and another VWA domain-containing protein (AMK58_03260). Proteins of another gene cluster consisting of a cytochrome C family protein (AMK58_03445), aldehyde dehydrogenase family protein (AMK58_03455), DUF779 domain protein (AMK58_03460), YVTN family beta propeller repeat protein (AMK58_03485), and cytochrome c-550 PedF protein (AMK58_03495) were also upregulated. Two proteins, PqqC (AMK58_06470) and PqqD (AMK58_06465), which are involved in PQQ biosynthesis, were upregulated along with two proteins of related function, PQQ-dependent dehydrogenase, methanol/ethanol family (AMK58_03515), and PQQ-dependent catabolism-associated CXXCW motif proteins (AMK58_03545). Another set of two adjacently encoded proteins, beta-ketoacyl-ACP synthase (AMK58_11130) and 3-oxoacyl-ACP reductase FabG (AMK58_11130), was upregulated. A global cell cycle regulator GcrA-like protein (AMK58_150153) and a STAS-domain anti-anti-sigma factor (AMK58_27140) were the two other proteins of interest that were detected only in *A. brasilense* Sp7 overexpressing RpoE6.

**TABLE 1:** List of upregulated proteins

Accession No.	Protein description	Gene symbol	Kegg ID	Abundance ratio (sample/control)
QEL94050.1	Flagellin	*fliC2*		63.195
QEL93526.1	Flagellar hook protein	*fliD*		2.197
QEL93527.1	Flagellar protein	*fliS*		100
QEL94060.1	Flagellar biosynthesis protein	*flgL*		100
QEL90617.1	Flagellar hook basal body complex protein	*fliE*		100
QEL94684.1	RNA polymerase sigma factor (RpoE8)		AMK58_24940	100
QEL94471.1	Hypothetical protein		AMK58_24945	100
QEL94472.1	Periplasmic heavy metal sensor		AMK58_24950	8.983
QEL90904.1	Cytochrome c family protein		AMK58_03445	3.627
QEL90902.1	Aldehyde dehydrogenase family protein		AMK58_03455	3.258
QEL90894.1	Cytochrome c-550 PedF		AMK58_03495	6.404
QEL90893.1	ABC transporter substrate-binding protein		AMK58_03500	7.436
QEL90890.1	PQQ-dependent dehydrogenase, methanol/ethanol family	*exaA*	AMK58_03515	8.35
QEL90886.1	PQQ-dependent catabolism-associated CXXCW motif protein		AMK58_03545	100
QEL90885.1	Cytochrome C		AMK58_03550	3.563
QEL89948.1	Pyrroloquinoline quinone biosynthesis protein	*pqqE*	AMK58_06460	5.083
QEL89950.1	Pyrroloquinoline quinone biosynthesis protein	*pqqC*	AMK58_06470	2.533
QEL89949.1	Pyrroloquinoline quinone biosynthesis peptide chaperon	*pqqD*	AMK58_06465	2.165

### The *fliC2* gene is co-transcribed with *fliD* but not with *flgL*

**Fig 2:**
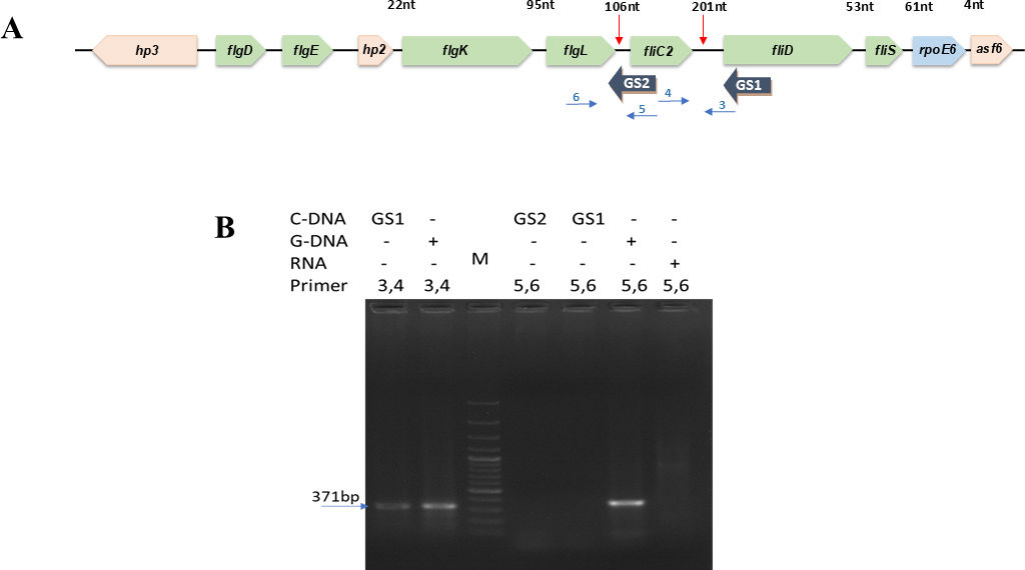
Genetic organization of the *rpoE6* cluster. (**A**) Organization of the genes encoding Hp3, FlgD, FlgE, Hp2, FlgK, FlgL, FliC2, FliD, FliS, RpoE6, and Asf6 in *Azospirillum brasilense* Sp7. The size of the intergenic regions between the genes and the locations of the primers used in 2B are indicated above the genes. (**B**) Co-transcription analysis of the *flgL, fliC2,* and *fliD* genes. The agarose gel shows the result of PCR amplifications. Lanes I and II show PCR amplification using primer sets 3 and 4, with cDNA transcribed using the GS2 primer and genomic DNA of *A. brasilense* Sp7, respectively, as templates, demonstrating that *fliC2* and *fliD* are co-transcribed. Lanes IV and V show no amplification using primer sets 5 and 6, with cDNA transcribed using primers GS1 and GS2, respectively, as templates, indicating the presence of a promoter region upstream of *fliC2*. Lane VI shows PCR amplification using primer sets 5 and 6 from genomic DNA as a positive control, and lane VII shows no amplification using primer sets 5 and 6 from purified DNase-treated RNA as a negative control. Primers 3, 4, 5, and 6 are primers FliDR, FliC2F, FliC2R, and FlgLF, respectively, as listed in Table S1. Lane V shows 1kb molecular weight marker (Thermo).

Since the genes encoding flagellar proteins FliC2, FliD, and FliS are upregulated by RpoE6 overexpression and are located upstream of *rpoE6*, we examined whether the genes located upstream of *rpoE6* are co-transcribed and form an operon with *rpoE6*. Since intergenic distances between *asf6-rpoE6*, *rpoE6-fliS,* and *fliS-fliD* are 4, 61, and 53 bp, respectively, the possibility of containing a promoter in these intergenic regions is rather low. But the distances between *fliD-fliC2* and *fliC2-flgL* of 201 and 106 bp, respectively, are large enough to accommodate a promoter upstream of *fliC2* or *fliD* (Fig. 2A). Since the intergenic distance between *fliD* and *fliC2* is rather large, we suspected the presence of a transcription terminator downstream of *fliD*. However, we did not find any transcription terminator in the intergenic region between *fliD* and *fliC2* using the ARNOLD server. We carried out a co-transcription analysis by RT-PCR using two sets of primers. One set of primers was complementary to *flgL* and *fliC2,* whereas the second set was complementary to *fliC2* and *fliD*. If either of the two pairs of primers leads to amplification of the desired amplicon size from the RNA, we presume that region is co-transcribed. But if an amplicon is not obtained, there may be a promoter in the intergenic region. Figure 2B shows the amplification of the expected size when primers 3 and 4 were used to amplify the intergenic region between *fliC2* and *fliD*, suggesting co-transcription and absence of a promoter in the intergenic region. On the other hand, the absence of amplification using primers 5 and 6 indicated an absence of co-transcription and the possible presence of a promoter in the upstream region of *fliC2*.

### The promoter driving the expression of *rpoE6* is located upstream of *fliC2*

**Fig 3:**
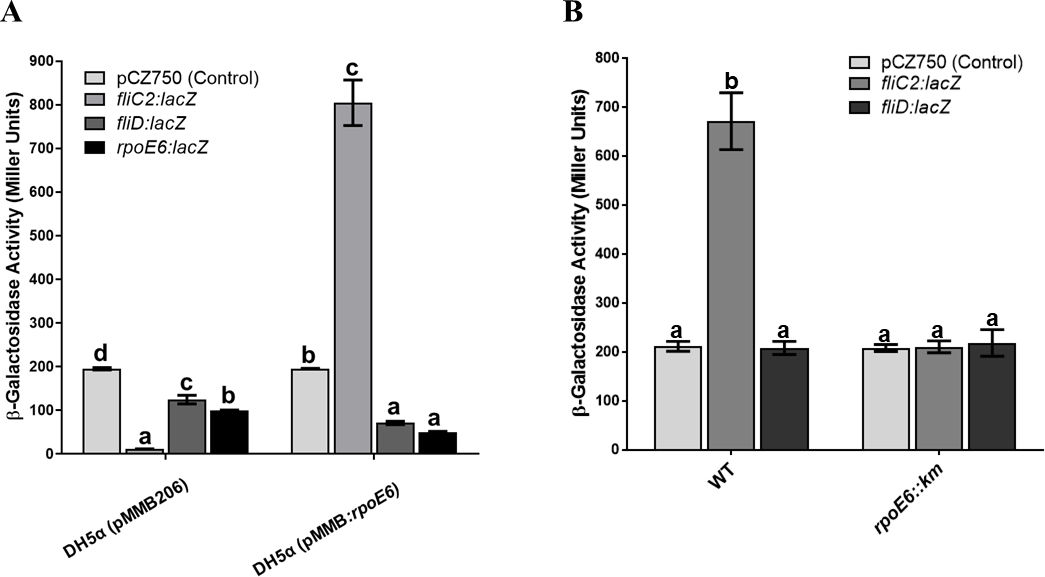
RpoE6 expression influences *fliC2* transcription. (**A**) Bar diagram showing β-galactosidase activity of *E. coli* DH5α (pMMB206) and DH5α (pMMB:*rpoE6*) harboring pCZ750 (plasmid vector control), *fliC2:lacZ*, *fliD:lacZ,* or *rpoE6:lacZ* fusion in LB culture medium after 6 h. (**B**) Bar diagram showing β-galactosidase activity of WT (*A. brasilense* Sp7) and its *rpoE6::km* mutant harboring pCZ750, *fliC2:lacZ,* or *fliD:lacZ* fusion after growth in minimal malate medium (MMM) for 6 h. Mean + SD of triplicates from three independent experiments is indicated, and differences between the mean values were compared. Lowercase letters above the bars represent different homologous subsets of different treatments after results of Duncan’s multiple comparison test, analyzed through SPSS software (different letters have *P* < 0.05).

To find a promoter upstream of *rpoE6*, we used a two-plasmid system in which RpoE6 was cloned in the pMMB206 under an IPTG-inducible promoter, and 500 bp upstream regions of *fliC2, liD,* and *rpoE6* were fused with a promoterless *lacZ* in pCZ750. The two plasmids were transferred into *E. coli* DH5α with the expectation that the expression of β-galactosidase activity would indicate an active promoter. Figure 3A shows that a conspicuously high level of β-galactosidase activity was observed in the *fliC2:lacZ* fusion, whereas it was negligible in the *rpoE6:lacZ* and *fliD:lacZ* fusions when RpoE6 was expressed, suggesting the presence of an active promoter in the *fliC2* upstream region and absence of a promoter in the upstream regions of *rpoE6* and *fliD*. This observation was further corroborated by examining the β-galactosidase activity from *fliC2:lacZ* and *fliD:lacZ* fusions in WT *A. brasilense* and *rpoE6::km*. In WT, the *fliC2:lacZ* fusion showed high expression, and this β-galactosidase activity was abolished in *rpoE6::km* (Fig. 3B). The *fliD:lacZ* fusion did not show β-galactosidase activity in WT nor *rpoE6::km*. This clearly shows that an RpoE6-recognizable promoter is present in the upstream region of *fliC2*, but not in *fliD*. This further indicates that *fliC2, fliD, fliS, rpoE6,* and *asf6* are likely transcribed as a single operon from a promoter located upstream of *fliC2*.

### Identification of transcription start site of *fliC2* by 5′ rapid amplification of cDNA end and effect of deletion of predicted −10 and −35 elements

**Fig 4:**
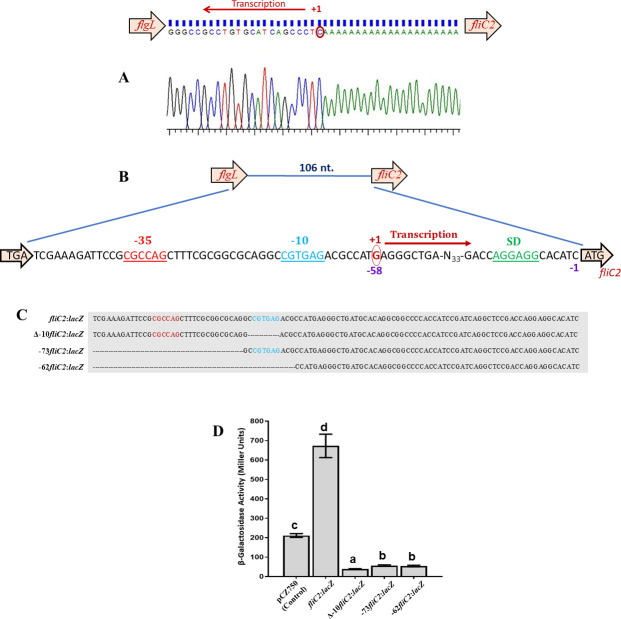
TSS and predicted −10 and −35 promoter elements of the *fliC2* promoter. (**A**) Intergenic nucleotide sequence (106 bp) between the stop codon of *flgL* and the start codon of *fliC2* in *A. brasilense* Sp7, showing TSS of *fliC2* based on the chromatogram determined by 5' RACE, in which "G," complementary to the encircled C, is indicated as the transcription start point. The chromatogram sequence is in reverse complement form. (**B**) The predicted Shine-Dalgarno (SD) sequence (AGGAGA) is highlighted in green and underlined. Predicted −10 element (CGTGAG) is shown in blue and underlined, and the −35 motif (CGCCAG), shown in red and underlined. The TSS is indicated by an encircled “G,” with the +1 position representing the first transcribed nucleotide. Nucleotide positions −1 and −58, marked in purple, indicate upstream regions relative to the *fliC2* start codon. (**C**) Graphical representation of the *fliC2* upstream region and its deletion derivatives fused to a promoterless *lacZ* reporter gene. The full-length construct (*fliC2:lacZ*) contains the intact native promoter. In the Δ–10 *fliC2:lacZ* construct, only the –10 promoter motif is specifically deleted, while the rest of the upstream sequence remains intact. The –73 *fliC2:lacZ* construct includes the region from –1 to –73, resulting in the deletion of upstream sequences that include the –35 element. The –62 *fliC2:lacZ* construct spans –1 to –62, deleting upstream sequences that remove both the –35 and –10 promoter elements. The –10 element (CGTGAG) is shown in blue, the –35 motif (CGCCAG) in red, and the TSS guanine (G) is highlighted in purple. (**D**) β-Galactosidase activity of Δ –10 *fliC2:lacZ*, –73 *fliC2:lacZ*, and –62 *fliC2:lacZ* fusions in *A. brasilense* Sp7 (WT) following 6-h growth in MMM. Mean + SD of triplicates from three independent experiments is indicated, and differences between the mean values were compared.

To identify the promoter elements of *fliC2*, we determined the transcription start site (TSS) of *fliC2* using 5′ rapid amplification of cDNA ends (5′ RACE). The TSS of *fliC2* was a “G” complementary to the encircled C, located 58 nucleotides upstream from the start codon (ATG) of FliC2 ORF. Based on the location of identified TSS, we predicted CGTGAG as the −10 element and CGCCAG as −35 element at a distance of 16 nucleotides (Fig. 4A and B). To examine whether similar promoters were present in other strains of *Azospirillum*, intergenic regions of 10 other strains/species of *Azospirillum* were aligned using ClustalW, which showed strong conservation of the sequence located at −10 and −35 elements. Notably, the −35 region was preceded by another conserved sequence CGCTTT (Fig. S2). A typical Shine-Dalgarno sequence (AGGAGA) was seven nucleotides upstream of the *fliC2* start codon. To ascertain whether the predicted −10 and −35 elements are functional, we cloned the upstream region of *fliC2* and its three derivatives (one having a deletion of −35 upstream sequence, the second lacking −10 sequence and the third lacking both −10 and −35 elements) with promoterless *lacZ* in pCZ750 vector (Fig. 4C). A comparison of the β-galactosidase activity of *A. brasilense* Sp7 harboring the four *lacZ* fusions showed that the derivatives lacking −10, −35, or both did not show any promoter activity, suggesting that the predicted −10 and −35 elements were essential for the promoter activity (Fig. 4D).

### Inactivation of the gene encoding RpoE6 affects multiple phenotypes in *A. brasilense* Sp7

**Fig 5:**
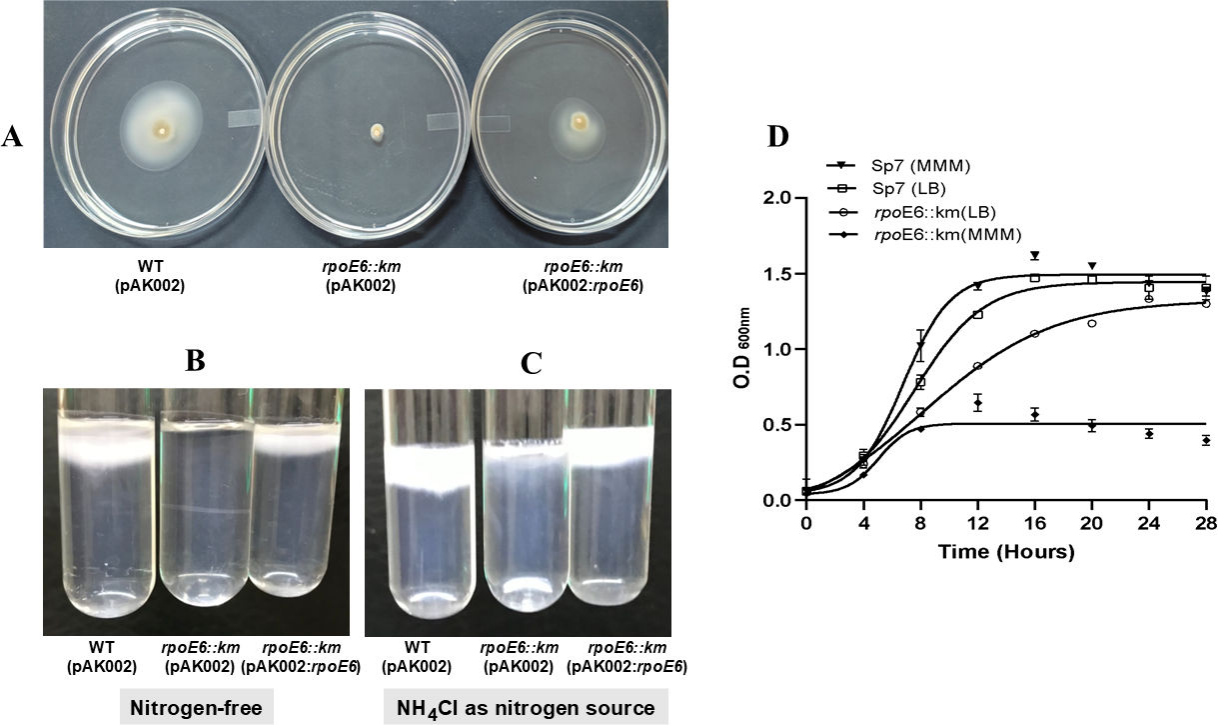
Motility and growth phenotypes of *rpoE6::km*. (**A**) Comparison of the swimming motility of WT (pAK002), *rpoE6::km* (pAK002), and *rpoE6::km* (pAK002:*rpoE6*) complemented strain on 0.25% MM agar plates, supplemented with 8 mM malate as the sole carbon source. (**B**) Comparison of pellicle formation ability of WT (pAK002), *rpoE6::km* (pAK002), and *rpoE6::km* (pAK002:*rpoE6*) in nitrogen-free semi-solid medium and (**C**) in semi-solid medium containing NH_4_Cl as the nitrogen source. (**D**) Comparison of the growth of WT (pAK002) and *rpoE6::km* (pAK002) in liquid MMM and LB media. Each point on the curve shows the mean value of the triplicates obtained from three independent experiments, and error bars at each point show standard deviation (SD).

Since proteins involved in flagellar filament biogenesis (including FliC2) were strongly upregulated by the overexpression of RpoE6, we suspected that RpoE6 would regulate the expression of FliC2 and some other flagellar filament proteins encoded nearby *rpoE6* on the genome. To investigate its role in flagellar motility, we inactivated the gene encoding RpoE6 by inserting a kanamycin resistance gene, and the WT copy of *rpoE6* was exchanged with the mutated copy via homologous recombination. The mutant (*rpoE6::km*), so obtained, showed drastically reduced swimming motility in 0.2% agar containing MMM (Fig. 5A).

Pellicle formation in an air gradient depends on swimming motility and the ability to navigate in air gradients in *A. brasilense*. Therefore, we compared pellicle-forming ability of *rpoE6::km* and its parent and found that *rpoE6::km* does not form any pellicle in nitrogen-free semi-solid medium (Fig. 5B) but shows relatively poor pellicle formation in semi-solid medium with NH_4_Cl as the nitrogen source (Fig. 5C), suggesting a severe but not null motility defect phenotype. In the above experiments, *rpoE6::km* expressing a cloned copy of *rpoE6* ectopically from a plasmid showed partial complementation of the swimming-related phenotype of the mutant.

**Fig 6:**
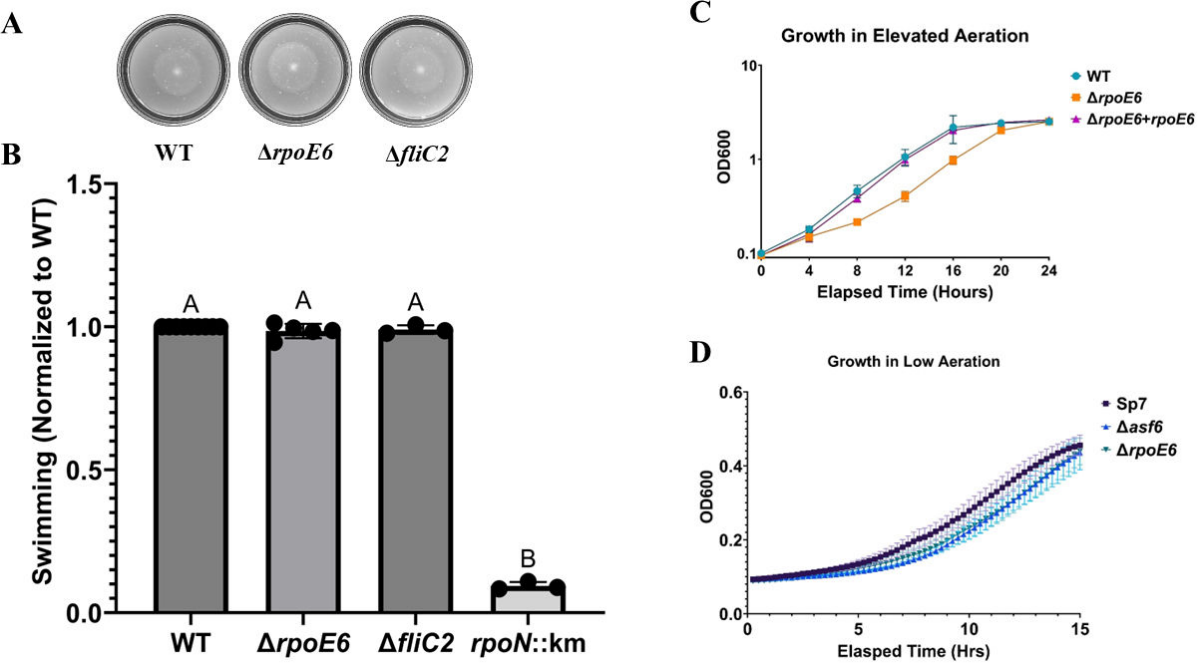
Motility and growth phenotypes of Δ*rpoE6* and Δ*fliC2*. (**A**) Representative images of *A. brasilense* Sp7 (WT), Δ*rpoE6,* and Δ*fliC2* in nitrogen-free soft agar (0.2% wt/vol) plate assays after 48-h incubation. (**B**) Quantitative analysis of soft agar plate assay in the same conditions mentioned in 5A. *rpoN::km* lacks polar and lateral flagella and was used as a negative control for motility. (**C**) Growth curves of WT with empty vector plasmid, Δ*rpoE6* with empty vector plasmid, and Δ*rpoE6* with the WT copy of rpoE6 expressed from the plasmid were measured over 24 h in 20 mL of MMAB media in 250 mL flasks for high aeration conditions. Measurements of OD_600_ were taken every 4 h. (**D**) Growth curves of WT and Δ*rpoE6* were measured every 20 min in 96-well plates for 15 h. Each point on the curve (**C and D**) shows the mean value of the triplicates obtained from three independent experiments, and error bars at each point show standard deviation (SD). The curves (**D**) appear smooth because the fitted lines connect closely spaced time-interval measurements.

**Fig 7:**
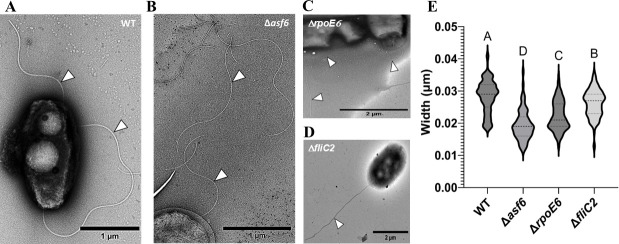
Flagellar structural phenotypes of WT, Δ*asf6*, Δ*rpoE6,* and Δ*fliC2*. (**A–D**) Representative TEM micrographs of each strain (**A**) WT, scale bar is 1 µm, (**B**) Δ*asf6*, scale bar is 1 µm, (**C**) Δ*rpoE6*, scale bar is 2 µm, and (**D**) Δ*fliC2,* scale bar is 2 µm. White arrows point to flagellar filaments. (**E**) The width of flagellar filaments was determined by measuring the distance between the outer black edges of the filaments and was analyzed in ImageJ software. Log-growing cells grown in MMAB media were used for this analysis. A two-way ANOVA test was used to determine statistical significance between strains. Different letters displayed above the plots indicate statistically significant distinct flagellar width. All strains were compared against each other.

To differentiate whether the reduced motility of *rpoE6::km* was due to the defect in flagellar biosynthesis or due to reduced growth, we compared the growth of *rpoE6::km* to the WT parent strain in liquid minimal medium and LB medium. The growth of *rpoE6::km* in the minimal medium was highly reduced in comparison to that in the LB medium (Fig. 5D). To clarify the motility and growth phenotypes of *rpoE6::km*, we constructed a new in-frame *rpoE6* mutant in which the entire *rpoE6* ORF was deleted and replaced with a chloramphenicol cassette (Δ*rpoE6*). This mutant was motile and chemotactic (Fig. 6A and B), indicating the initial mutation was polar on downstream genes, an effect that would also explain the partial complementation observed above. The Δ*rpoE6* mutant retained a reduced growth rate when incubated under elevated aeration relative to the WT or a complemented strain (Fig. 6C), but not significantly when incubated with low shaking (Fig. 6D), indicating that the defective growth under high aeration is associated with the *rpoE6* mutation. We also observed that the non-polar Δ*rpoE6* mutant produced thinner filaments compared to WT (Fig. 7).

### Inactivation of the gene encoding FliC2 does not affect swimming

Although orthologs of FliC2 in *A. brasilense* are found in several other bacteria, their role in flagellar biosynthesis and swimming motility is not known. Unlike other flagellins encoded in the *A. brasilense* Sp7 genome, FliC2 NCBI Locus: WP 035682079; Gene Locus Tag: OH82_RS01130; a homolog of AZOBR_ p340061 is the smallest (274 aa) flagellin. FliC2 shows 84%, 57.6%, and 56% identity with its putative orthologs in *A. lipoferum*, *R. palustris,* and *B. japonicum*, respectively, which are also of 274 aa (Fig. S3). Its ortholog FlaA is also found in *Bradyrhizobium diazoefficiens* as blr3695.

To examine the role of FliC2 in swimming, we deleted the ORF coding for FliC2 and replaced it with a chloramphenicol resistance cassette, yielding an in-frame deletion (strain Δ*fliC2*). Mutating the gene did not affect swimming (Fig. 6A and B). We also did not detect a difference between the WT and the Δ*fliC2* mutant when examining cells by TEM, though the mutant flagellar filament thickness was slightly reduced compared to the parent, suggesting a change in its structure (Fig. 7). Together, the data suggest that FliC2 has a minor role in motility and flagellum structure.

### FliC2 flagellin is absent in flagellar protein extracts of mutants lacking *rpoE6* or *fliC2*

**Fig 8:**
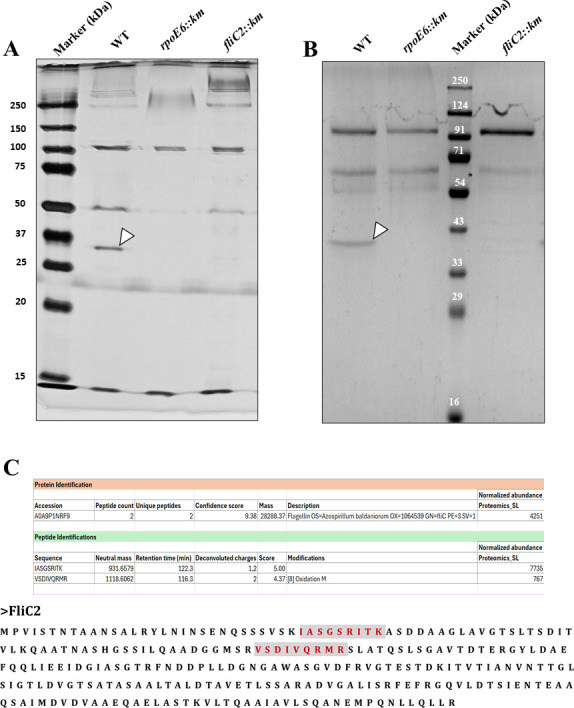
Extracellular proteins obtained from the supernatant of *rpoE6::km*, *fliC2::km,* and *A. brasilense* Sp7. (**A**) The extracellular proteins were separated by SDS-PAGE and stained with silver stain and (**B**) Coomassie brilliant blue. The band representing the FliC2 flagellin is absent in flagellar protein extracts of *rpoE6::km* and *fliC2::km* but is present in WT, and its position is indicated by an arrow. (**C**) UPLC-MS^E^ results showing predicted mass and sequence of the two peptides produced from the band predicted to be FliC2. Given below is the full amino acid sequence of FliC2 of *A. baldaniorum* showing the two peptides (marked in red and highlighted in gray) identified by UPLC-MSE analysis.

If our inference that the expression of FliC2 is regulated by RpoE6 is true, then we presume that mutants lacking *rpoE6* will lack the expression of FliC2. To analyze this, we compared the extracellular protein profiles of *fliC2::km* and *rpoE6::km* with those of WT. We observed a low molecular weight protein present in the WT but missing in *rpoE6::km* and *fliC2::km* (Fig. 8A and B), suggesting that the low molecular weight protein could correspond to FliC2. We also observed that sometimes this low molecular weight protein migrated at ~ 28kDA, while other times, it migrated at around 35 kDa (Fig. 8A and B). The FliC2 predicted molecular weight is 28 kDa, suggesting one of the bands is FliC2. Since the large polar flagellin of *A. brasilense* is glycosylated (31), we suspected the two bands could represent an unmodified and a modified FliC2 protein. To ascertain their identity, the protein bands were subjected to ultra-performance liquid chromatography (UPLC)-MS^E^ after proteolysis, and the peptide mass of the two peptides (IASGSRITK and VSDIVQRMR) matched with the amino acid sequence present in the FliC2 protein of *A. baldaniorum* (Fig. 8C). It is likely that unknown variable(s) in our experimental conditions affect FliC2 modification. We surmise that FliC2 may be glycosylated, like the large polar flagellin.

### Asf6 and RpoE6 together modulate the abundance of FliC2

**Fig 9:**
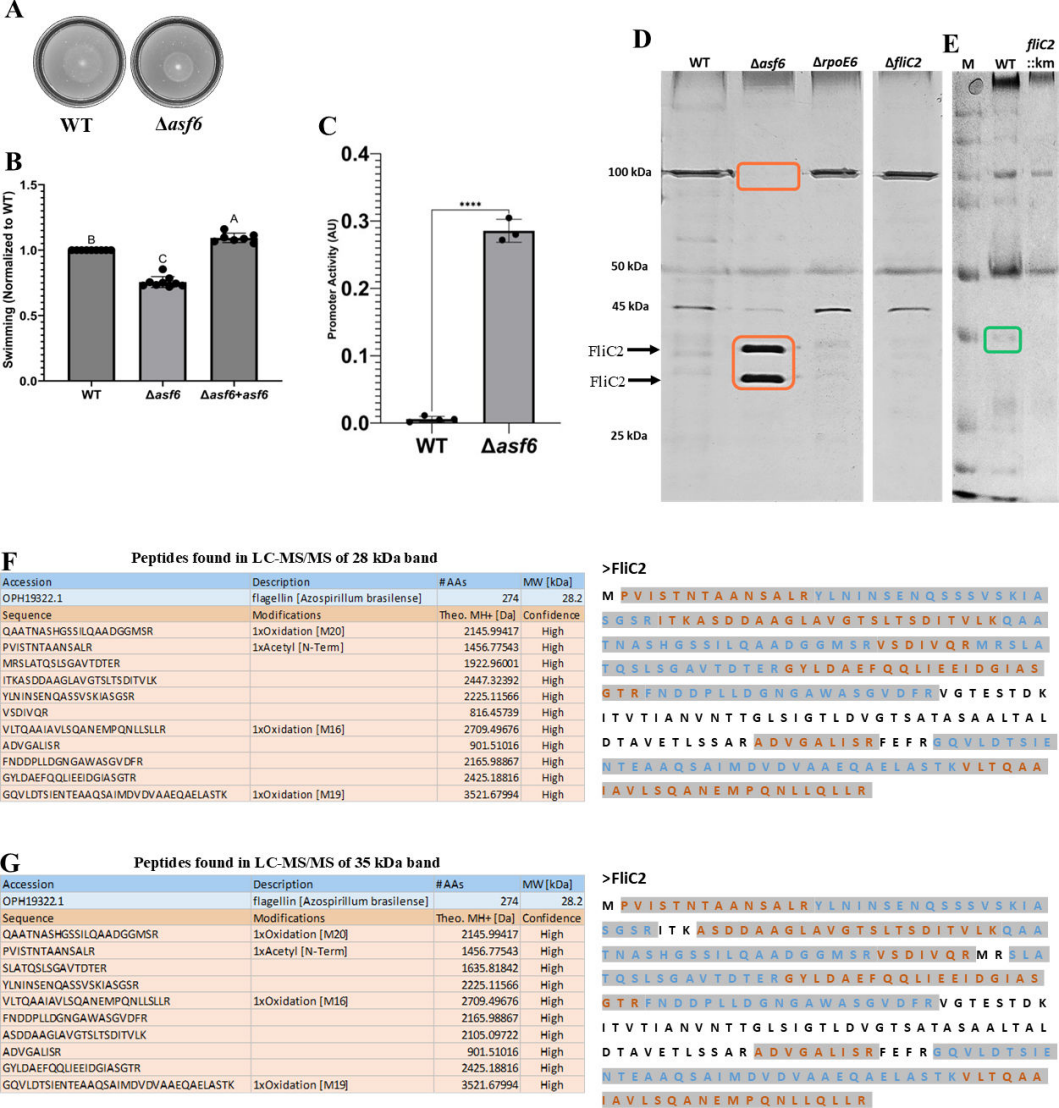
Δ*asf6* motility and FliC2 expression. (**A**) Representative images of *A. brasilense* Sp7 (WT) as seen in Fig. 5A, and Δ*asf6* in nitrogen-free soft agar (0.2% wt/vol) plate assays. Both strains form chemotactic rings indicative of motility after 48-h incubation. (**B**) Quantitative analysis of soft agar swimming normalized to the WT. WT and Δ*asf6* both carry an empty vector, while the complemented strain (Δ*asf6 + asf6*) carries a WT *asf6* expressed from the plasmid. (**C**) Promoter activity of *fliC2* in WT and ∆*asf6* using a β-glucuronidase assay. (**D**) ∆*asf6* produces high amounts of FliC2 protein in a silver-stained SDS-PAGE. Two bands may reflect different glycosylation patterns. (**E**) PAS-Schiff stain shows that the band suspected to be FliC2 is glycosylated. (**F**) LC-MS/MS results showing predicted protein ID and sequences of peptides found from the silver-stained band at 28 kDa in ∆*asf6*. Sequences of peptides found are shown as gray-highlighted parts of the protein sequence of FliC2. Orange and blue colors are used to separate peptides following each other in the sequence. (**G**) LC-MS/MS results showing predicted protein ID and sequences of peptides found from the silver-stained band at 35 kDa in ∆*asf6*. The sequence of FliC2 is shown with the same annotation as the previous panel. "****" indicates a statistically highly significant difference (*P* < 0.0001)

While comparing the gene organization of *rpoE6* in *A. brasilense* Sp7 and its orthologs in other strains of *A. brasilense* and other species of *Azospirillum,* such as *A. thiophilum* and *A. lipoferum*, we noted the presence of a gene encoding a small hypothetical protein of 98 aa immediately downstream of *rpoE6* that we have named anti-sigma factor (*asf6*) (see below). A deletion of *asf6* (D*asf6*) yielded a mutant that has a defect in motility relative to WT (Fig. 9A and B). The motility defect of Δ*asf6* is fully complemented by expressing the WT *asf6* gene *in trans* from a plasmid (Fig. 9B). We next compared the flagella produced by this mutant by TEM and found that the mutant lacking *asf6* produced flagellar filaments that were thinner than those produced by the WT (Fig. 7).

The proteomic data (Table 1) show that FliC2 is upregulated when RpoE6 is overexpressed. The *fliC2* gene is also encoded in close proximity to *rpoE6* and *asf6*. Due to these observations, we hypothesized that RpoE6 is transcriptionally upregulating *fliC2* to increase the abundance of the FliC2 protein within the polar flagellum filament. To test this, we constructed a *fliC2* promoter fusion to a promoterless β-glucuronidase (*gusA*) gene expressed from a broad host range vector and tested it in the WT and the Δ*asf6*. As expected, *fliC2* promoter activity was negligible in the WT background, suggesting that *fliC2* is not highly expressed under the conditions tested. However, in Δ*asf6*, the *fliC2* promoter activity was high (Fig. 9C), suggesting that the absence of Asf6 results in the elevation of transcription of *fliC2*. To further characterize the effect on flagellar filament structure, we analyzed extracellular proteins produced by the WT and compared these with those produced by the Δ*asf6*, Δ*rpoE6,* and Δ*fliC2* mutant strains (Fig. 9D). We found that the Δ*asf6* strain produced two protein bands (~28 kDa and ~35 kDa) that were not detected in the Δ*rpoE6* and Δ*fliC2* mutants but faint (~35 kDa band only) in the WT (Fig. 9D and 8B). Mass spectrometry analysis of excised bands using Nano LC-MS/MS confirmed that both bands corresponded to FliC2 (Fig. 9F and G). We also used a PAS-Schiff stain on extracellular protein extracts and found that the protein migrating at ~35 kDa, but not the protein migrating at ~28 kDa, stained in the WT strain but not in *fliC2::km* (Fig. 9E). Together, the results indicated that the ~35 kDa protein is likely a glycosylated form of FliC2. We also noted that the extracellular protein extracts of the Δ*asf6* mutant lacked the ~100 kDa band corresponding to the polar flagellin, but the other mutant strains analyzed did not (Fig. 9D). These observations suggest that the mutant lacking *asf6* produces a flagellar filament that likely contains almost exclusively FliC2, which could explain the structural changes observed from TEM images (Fig. 7).

### Asf6 interacts with RpoE6 in the bacterial two-hybrid assay

**Fig 10:**
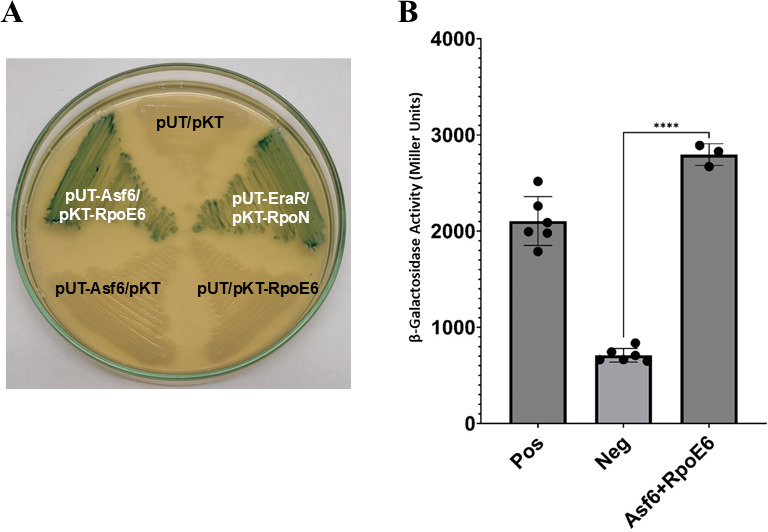
Protein-protein interaction between RpoE6 and Asf6 assessed using the BacTH system. (**A**) *Escherichia coli* BTH101 cells were co-transformed with plasmid pairs including pUT/pKT, pUT-EraR/pKT-RpoN, pUT/pKT-RpoE6, pUT-Asf6/pKT, and pUT-Asf6/pKT-RpoE6. Interactions between RpoE6 and Asf6 were assessed qualitatively by streaking transformed colonies on LB agar plates supplemented with 0.5 mM IPTG and 40 μg/mL X-Gal. Blue coloration indicated positive interactions. The plasmid combinations pUT/pKT, pUT/pKT-RpoE6, and pUT-Asf6/pKT served as negative controls, while pUT-EraR/pKT-RpoN (34) was used as a positive control. (**B**) The protein-protein interaction of Asf6 and RpoE6 was quantified by calculating Miller Units using the BacTH assay. Mean + SD of triplicates from three independent experiments is indicated, and differences between mean values are compared. The positive control used was pUT18-Zip + pKT25 Zip, two proteins known to interact.

The intergenic distance between the genes encoding RpoE6 and Asf6 is only 4 bp, and these genes might be translationally coupled (Fig. 2A). Furthermore, Asf6 and RpoE6 have opposite effects on FliC2 abundance. Therefore, we next tested whether Asf6 and RpoE6 could physically interact in a bacterial two-hybrid assay (BacTH). In the BacTH assay, protein-protein interactions are determined by the reconstitution of adenylate cyclase subdomains T18 and T25 to the functional holoenzyme. When Asf6 (cloned in pKT25 or pUT18) was expressed individually in *E. coli* BTH101 cells, it did not show any β-galactosidase activity, indicating no self-activation of the construct tested. Co-expression of Asf6 and RpoE6, whether from pKT25 or pUT18, showed the same high level of β-galactosidase activity (Fig. 10A and B), indicating a positive protein-protein interaction between Asf6 and RpoE6. Quantitative evaluation confirmed the interactions (Fig. 10B).

### RpoE6 is required for the growth of *A. brasilense* Sp7 on fructose and glycerol

**Fig 11:**
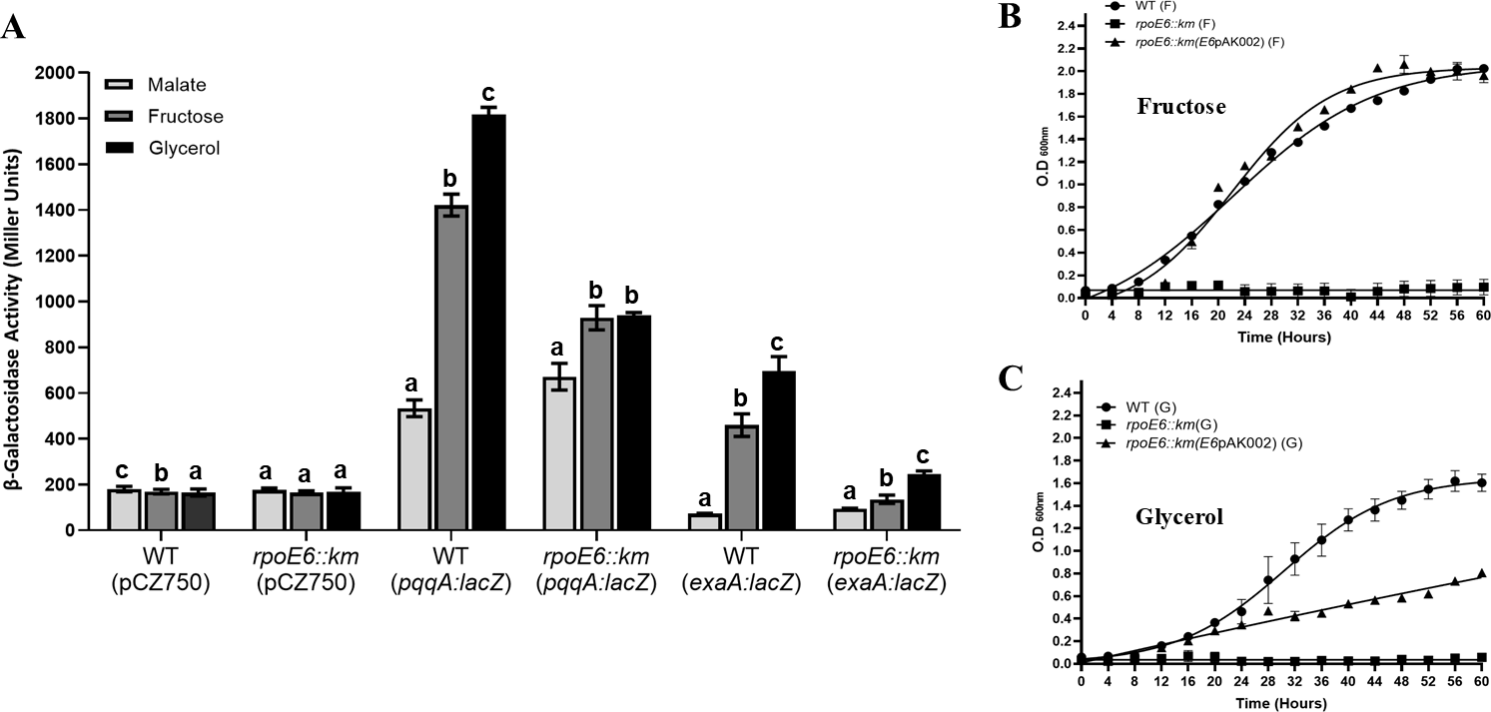
Metabolism of fructose and glycerol in *rpoE6::km*. (**A**) Effect of different carbon sources (malate, fructose, and glycerol, each at 40 mM) on β-galactosidase activity from the pCZ750 (plasmid vector control), *pqqA:lacZ*, and *exaA:lacZ* fusions in WT (*A. brasilense* Sp7) and its *rpoE6::km* mutant, grown for 6 h. Mean + SD of triplicates from three independent experiments is indicated, and differences between mean values are compared. Lowercase letters above the bars represent different homologous subsets of different treatments after results of Duncan’s multiple comparison test, analyzed through SPSS software (different letters have *P* < 0.05). Growth curve of the WT, *rpoE6::km* mutant, and *rpoE6::km* (pAK002:rpoE6) complemented strain in (**B**) minimal fructose medium (MFM, containing 40 mM fructose as the carbon source) and in (**C**) minimal glycerol medium (MGM, containing 40 mM glycerol as the carbon source). Each point on the curve shows the mean value of the triplicates obtained from three independent experiments, and error bars at each point show standard deviation (SD).

From the proteomic data (Table 1), we noted that proteins involved in the biosynthesis of PQQ and PQQ-dependent alcohol dehydrogenase were upregulated by RpoE6 overexpression. To validate this observation further, we constructed *exaA:lacZ* and *pqqA:lacZ* fusions and examined their expression in WT and *rpoE6::km*. We found that the expression of *exaA:lacZ* and *pqqA:lacZ* fusions was upregulated by fructose and glycerol in *A. brasilense* Sp7 but highly compromised in the *rpoE6::km* (Fig. 11A). Since PQQ and PQQ-dependent alcohol dehydrogenase (ExaA) are required for the growth of *A. brasilense* Sp7 on glycerol and fructose (34, 35), we compared the ability of *rpoE6::km* and *A. brasilense* Sp7 to grow on fructose or glycerol as the sole source of carbon. Figure 11B and C shows that *rpoE6::km* is highly compromised in its ability to use fructose (Fig. 11B) and glycerol (Fig. 11C) for growth. Expression of the WT copy of *rpoE6* in *rpoE6::km* restored the ability to grow on fructose almost completely and, to a large extent, also to grow on glycerol. These results showed that RpoE6 is required for the optimal growth of *A. brasilense* Sp7 on fructose and glycerol and for maximal induction of promoters for *exaA* and *pqq* genes involved in glycerol and fructose metabolism.

### RpoE6 regulates the expression of RpoE8

**Fig 12:**
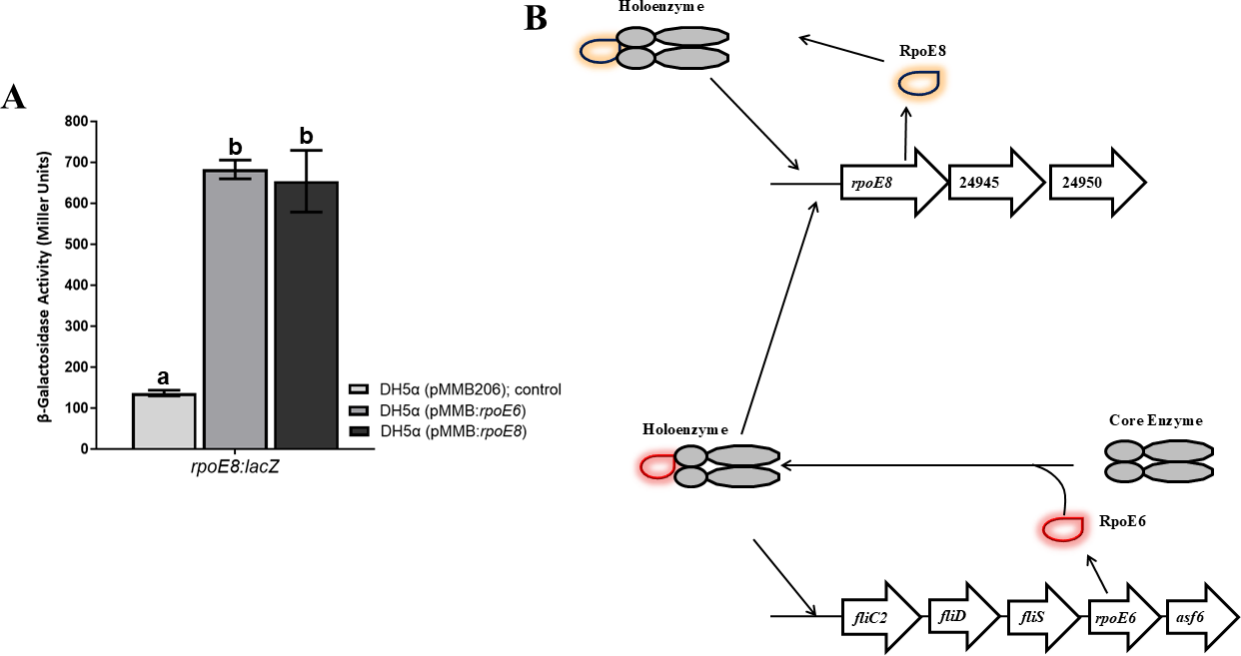
Regulation of *rpoE8* by RpoE6. (**A**) Bar diagram showing β-galactosidase activity from *rpoE8:lacZ* fusion in *E. coli* DH5α (pMMB206), DH5α (pMMB:*rpoE6*), and DH5α (pMMB:*rpoE8*) grown in LB medium for 6 h. Mean + SD of triplicates from three independent experiments is indicated, and differences between the mean values were compared. Lowercase letters above the bars represent different homologous subsets of different treatments after results of Duncan’s multiple comparison test, analyzed through SPSS software (different letters have *P* < 0.05). (**B**) Model based on our hypothesis of RpoE6 regulating the expression of *fliC2*, *fliD*, *fliS*, *rpoE6,* and *asf6* from the *fliC2* promoter, as well as the RpoE6-driven expression of *rpoE8* and its two downstream genes in a tricistronic operon with RpoE8 auto-activating its transcription.

The proteomic data shown in Table 1 identified three proteins encoded by AMK58_24940, AMK58_24945, and AMK58_24950, including RpoE8, that were upregulated by overexpression of RpoE6. To validate this observation, we used a *rpoE8:lacZ* fusion and mobilized it into *E. coli* DH5*α*. Then, we expressed the WT copy of *rpoE6* and *rpoE8* on a compatible plasmid and observed the effect on the expression of the *rpoE8:lacZ* fusion (Fig. 12A). The upregulation of β-galactosidase activity of *rpoE8:lacZ* fusion by *rpoE6* and *rpoE8* expression suggests that the *rpoE8* promoter is regulated by RpoE6 as well as by RpoE8 itself. Based on the results obtained in this study, we conclude that RpoE6 regulates the expression of *fliC2*, *fliD*, *fliS*, *rpoE6,* and *asf6* from the *fliC2* promoter, and of *rpoE8* and the following two genes of a tricistronic operon (Fig. 12B). At this time, we do not yet know what the function of the tricistronic *rpoE8* operon is.

## DISCUSSION

Here, we report that the alternative sigma factor RpoE6 is involved in regulating some polar flagellar biosynthesis genes as well as genes involved in metabolism and a gene coding for another sigma factor, RpoE8. Therefore, RpoE6 has a distinct role compared to that of the enteric FliA. Our findings indicate that RpoE6 has a regulatory role that seems to couple expression of FliC2 with induction of glycerol and fructose catabolism, as well as uncharacterized genes controlled by RpoE8. That RpoE6 regulates another sigma factor, which is not unexpected: alternative sigma factors, such as RpoE6, are known to form regulatory cascades in which one sigma factor regulates the expression of another sigma factor (36, 37). Therefore, RpoE6 appears to function within a larger and more complex regulatory cascade that couples metabolism and flagellar filament structure and function. We are not aware of a similar function in other bacteria studied to date. However, the genomic organization of RpoE6 homologs in other Alphaproteobacteria suggests this function may be widespread in phylogenetically related bacteria.

RpoE6 regulates genes encoding proteins comprising the flagellar hook-filament junction (FlgK and FlgL) and the flagellar filament (FliC2, FliS, and FliD). Conservation of synteny in the organization of the *rpoE6* gene cluster in the different species of *Azospirillum* and the occurrence of a similar organization in other Alphaproteobacteria suggest that RpoE6 and the proteins encoded upstream might be involved in a common function related to the biogenesis of flagella. The presence of RpoE6 homologs in *R. palustris*, *M. gryphiswaldense,* and *B. japonicum,* showing >50% identity, also located in the vicinity of the genes involved in the formation of flagellar filament, suggests a similar role for this alternative sigma factor in these members of Alphaproteobacteria. In *B. diazoefficiens* (strain: 110spc4), *fliC* (Bdiaspc4_RS19025), *fliD* (Bdiaspc4_RS19030), and *fliS* (Bdiaspc4_RS19035) are also located downstream of a gene encoding an RNA polymerase sigma factor (Bdiaspc4_RS19020). But the role of neither FliC nor the ECF sigma factor has been described.

Although small flagellins similar to the FliC2 of *A. brasilense* Sp7 have been reported previously in other bacteria, their role in the motility and formation of the flagellar filament was not described. Data obtained here indicate that mutants lacking FliC2 have no detectable swimming defect but appear to produce a flagellar filament with a minor alteration of its structure compared to the WT (as indicated in the thickness measurements), suggesting a minor role for the flagellin in *A. brasilense* polar flagellum structure and function. In a mutant lacking Asf6, the polar filament was likely to be almost exclusively comprised of FliC2, and the flagellum also appears to have a different structure (as indicated in the thickness measurements) compared to WT. The regulation of *fliC2* and associated flagellar filament and hooks encoded proteins described here suggests that the abundance of FliC2 within the polar flagellum filament is inversely modulated depending on the activity of RpoE6 and of Asf6. Together, these data indicate that FliC2 may be a minor flagellin component of the polar flagellum and that its overexpression can alter the structure of the flagellum and likely, its function. The nature of signal(s) that activates RpoE6 and the exact impact of FliC2 abundance on polar flagellum function remain to be identified. Our findings further imply that the abundance of the FliC2 minor flagellin within the polar flagellar filament alters the function of this appendage. In *Shewanella putrefaciens,* the flagellin composition of the filament affects the polar flagellar morphology and its function to adapt to swimming under different conditions (38). The exact role that increased FliC2 abundance in the *A. brasilense* flagellum may have on flagellum function and on cells swimming remains to be characterized.

Lack of Asf6 reduced the abundance of the polar flagellin while increasing that of FliC2, whereas lack of RpoE6 also appeared to reduce the abundance of the polar flagellin. Both mutants have distinct polar flagellum filament structures. We do not yet understand how the lack of Asf6 or RpoE6 affects polar flagellin abundance, but these observations suggest there may be some feedback between the Asf6-RpoE6 network and polar flagellin biosynthesis and/or stability. It is likely that the effects of the RpoE6 mutation on metabolism are related to these changes.

Flagellins show considerable variation in their size. All the different types of flagellins show conservation in the amino acid residues in the D0 and D1 domains at the N- and C-termini and variability in the D2 and D3 domains of the central regions (39, 40). In this study, we found that FliC2 (28 kDa) possesses only D0 and D1 domains and lacks D2 and D3 domains (Fig. S4, and is considerably smaller than the polar flagellin previously identified in *A. brasilense* (9). However, we also obtained evidence supporting that FliC2, like the major polar flagellin, is likely glycosylated and contributes to flagellum structure and function. Glycosylation of FliC2 would likely support its incorporation into the filament produced by the more abundant glycosylated polar large flagellin. Occurrence of more than one type of flagellins is not uncommon in the bacterial world as flagellar filaments of other Alphaproteobacteria, such as *Rhizobium lupine* H13-3, *Sinorhizobium meliloti*, *Rhizobium leguminosarum* (41), and *Agrobacterium tumefaciens* (42) consist of 3–4 types of flagellins (43). In *B. japonicum,* two sets of flagella: one thick polar flagellum and a few thin sub-polar flagella were reported. The thin flagella also consist of two flagellins (FliCI and FliCII) (44). Similarly, *Caulobacter crescentus* flagella consist of six flagellins (45). In most cases, the role that multiple flagellins have on flagellum function is not fully understood.

We also found that an anti-sigma factor, Asf6, interacts with RpoE6 and likely antagonizes its activity in the regulation of motility. The following lines of evidence suggest that Asf6 is an anti-sigma factor of RpoE6: (i) Asf6 and RpoE6 physically interact, (ii) Asf6 reduces the activity of RpoE6 (deletion of *asf6* leads to overproduction of FliC2), and (iii) Overexpression of RpoE6 reduces the impact of Asf6 on FliC2 expression (i.e., FliC2 abundance increases relative to WT). In this respect, RpoE6 may resemble the enteric FlgM-FliA interaction (22). However, given the other effects that RpoE6 has on metabolism (see below), RpoE6 is unlikely to function similarly to FliA since it has additional gene targets other than those encoding flagellar proteins.

Expression of RpoE6 seems to cause the positive regulation of proteins that are involved in various uptake and metabolic processes, including genes implicated in the catabolism of glycerol and fructose, such as *exaA* and *pqqA,* which are required for the growth of *A. brasilense* Sp7 on fructose and glycerol as carbon sources (34). In *A. brasilense,* growth on fructose induced the expression of FliC2 (46), confirming the link between fructose metabolism and FliC2 expression. Fructose and glycerol catabolism allow *A. brasilense* to co-metabolize ethanol, which may be present when plant roots are waterlogged, and root respiration is interrupted (35). Interestingly, growth on fructose also induces a type 6 secretion system that functions to kill other bacterial species and induces the production of a black pigment (46). These observations, combined with our data, could suggest that RpoE6 functions within a regulatory cascade that facilitates metabolic and physiological adaptation of *A. brasilense* to unfavorable conditions related to fructose metabolism. However, the exact nature of these conditions remains to be identified.

## MATERIALS AND METHODS

### Bacterial strains, plasmids, and chemicals

The *E. coli* strains DH5α, S17.1, and BTH101 were maintained and grown in LB medium at 37°C. *A. brasilense* Sp7 and its different mutants (*rpoE6::km, fliC2::km*) were grown in MMM as having 40 mM malate as a sole carbon source and were maintained at 30°C (47). All chemicals used for culturing bacterial strains were from Merck KGaA (Darmstadt, Germany), Sigma-Aldrich Corporation (St. Louis, USA), and Hi-media (Mumbai, India). The enzymes used for cloning were purchased from New England Biolabs. The strains and plasmids used in this work are listed in Table 2, and primers used are listed in Table S1.

**TABLE 2:** Bacterial strains and plasmids

Strains and plasmids	Relevant properties	References/sources
Bacterial strains		
*A. brasilense* Sp7	Wild-type strain (ATCC 29729)	(48)
*E. coli* DH5α	Δ*lacU169, hsdR17, recA1, endA1, gyrA96, thiL, relA1*	Gibco/BRL
*E. coli* S.17-1	Sm^r^*, recA, thi, pro, hsdR,* RP4-2(Tc::Mu; Km:: Tn*7*)	(49)
*E. coli* BTH101	F^−^*cya-99 araD139 galE15 galK16 rpsL1* (Str^r^) *hsdR2 mcrA1 mcrB1*	Eurumedex
*rpoE6::km* strain	*A. brasilense* Sp7 derivative containing Km^r^ cassette in place of nucleotides *+*760 to +1,130of the *rpoE6* gene.	This work
*fliC2::km* strain	*A. brasilense* Sp7 derivative containing Km^r^ cassette in place of nucleotides *+*100 to +743 of the *fliC2* gene.	This work
Δ*rpoE6*	*A. brasilense* Sp7 derivative with chloramphenicol-resistant cassette replacement of the deleted *rpoE6* gene.	This work
Δ*asf6*	*A. brasilense* Sp7 derivative with chloramphenicol-resistant cassette replacement of the deleted *asf6* gene.	This work
Δ*fliC2*	*A. brasilense* Sp7 derivative with chloramphenicol-resistant cassette replacement of the deleted *fliC2* gene.	This work
Plasmids		This work
pSUP202	ColE1 replicon, mobilizable plasmid, suicide vector suitable for *A. brasilense*; Amp^r^, Tc^r^, Cm^r^	(49)
pUC4K	Vector containing the Km^r^ cassette	GE Healthcare
pMMB206	Cm^r^, broad host range, low-copy-number expression vector	(50)
pKT25N	Encodes T25 fragment (corresponding to the first 224 amino acids of CyaA) with *lac* promoter; derived from low-copy-number plasmid pSU40; Km^r^; expresses chimeric proteins	Eurumedex
pUT18C	pUT18 derived from vector pUC19; Amp^r^; encodes the T18 fragment (amino acids 225–399 of CyaA) with *lac* promoter; it expresses chimeric proteins	Eurumedex
pCZ750	pFAJ1700 containing the KpnI-AscI *lacZ* gene from pCZ367 plasmid; Tc^r^, Amp^r^	(51)
pAK002	pBBR1MCS-3 derivative containing the constitutive kanamycin resistance gene (*apt*) promoter	(28)
pSUP:*rpoE6*	*rpoE6* gene disrupted by Km^r^ cassette cloned into the EcoRI-Pstl site in pSUP202	This work
pSUP:*fliC2*	*fliC2* gene disrupted by Km^r^ cassette cloned into the EcoRI-Pstl site in pSUP202	This work
pAK002:*rpoE6*	*rpoE6* gene from *A. brasilense* Sp7 cloned into the XhoI restriction site of pAK002	This work
*fliC2*:*lacZ*	pCZ750 derivative; Tc^r^, Amp^r^, *fliC2:lacZ*,	This work
*fliD:lacZ*	pCZ750 derivative; Tc^r^, Amp^r^, *fliD:lacZ*,	This work
*rpoE6:lacZ*	pCZ750 derivative; Tc^r^, Amp^r^, *rpoE6:lacZ*,	This work
*exaA:lacZ*	pCZ750 derivative; Tc^r^, Amp^r^, *exaA:lacZ*,	(34)
*pqqA*:*lacZ*	pCZ750 derivative; Tc^r^, Amp^r^, *ppqA:lacZ*,	This work
*rpoE8*:*lacZ*	pCZ750 derivative; Tc^r^, Amp^r^, *rpoE8:lacZ*,	This work
Δ –10 *fliC2:lacZ*	pCZ750 derivative; Tc^r^, Amp^r^, *fliC2:lacZ* lacking −10 element (−73 to −66)	This work
–73 *fliC2:lacZ*	pCZ750 derivative; Tc^r^, Amp^r^, *fliC2:lacZ* with deletion of 33 bp (−106 to −74)	This work
–62 *fliC2:lacZ*	pCZ750 derivative; Tc^r^, Amp^r^, *fliC2:lacZ* with deletion of 44 bp (−106 to −62)	This work
pKT25N:*rpoE6*	*rpoE6* gene was cloned at the XbaI and EcoRI sites of the pKT25 vector	This work
pUT18C:*asf6*	*asf6* gene was cloned at the XbaI and EcoRI sites of the pUT18C vector	This work
PFUS1-*fliC2*	*fliC2* gene cloned at HindIII and EcoRI sites of the pFUS1 vector	This work
pK18mobsacB- *rpoE6-*Cm	Upstream of the *rpoE6* gene + Cmr cassette + downstream of the *rpoE6* gene cloned at the Xba1 and HindIII sites of the pK18mobsacB vector	This work
pK18mobsacB- *asf6*-Cm	Upstream of the *asf6* gene + Cmr cassette + downstream of the *asf6* gene cloned at the Xba1 and HindIII sites of the pK18mobsacB vector	This work
pK18mobsacB-*fliC2*-Cm	Upstream of the *fliC2* gene + Cmr cassette + downstream of the *fliC2* gene cloned at the Xba1 and HindIII sites of the pK18mobsacB vector	This work

### Bioinformatics analysis and gene organization

To construct the genetic map of *rpoE6*, its genomic organization was examined by analyzing the arrangement of its flanking gene. The sequence of RpoE6 orthologs from other bacteria was retrieved from the NCBI database (https://www.ncbi.nlm.nih.gov/) while the amino acid sequences of FliC2, Asf6, and RpoE8 of *A. brasilense* Sp7 were retrieved from the KEGG database (https://www.genome.jp/kegg/). Percentage sequence identity and sequence similarity were calculated by using BLAST (https://blast.ncbi.nlm.nih.gov/Blast.cgi), and alignment of sequences was performed by the ClustalW program of BioEdit software.

### Insertional inactivation and in-frame deletion of *rpoE6, fliC2,* and *asf6* in *A. brasilense* Sp7

For insertional inactivation of *rpoE6, fliC2,* and *asf6* genes, upstream and downstream regions of each gene (∼1,000 bp) were PCR-amplified in two parts as Amplicons A and B by using a specific set of primers having EcoRI/Bglll and Pstl/Bglll restriction sites. Briefly, Amplicon A was amplified using primer sets RpoE6_AFP and RpoE6_ARP (for gene *rpoE6*), FliC2_AFP and FliC2_ARP (for gene *fliC2*), and with ASF6_AFP and ASF6_ARP (for gene *asf6*). Similarly, Amplicon B was amplified using primer sets RpoE6_BFP and RpoE6_BRP (for gene *rpoE6*), FliC2_BFP and FliC2_BRP (for gene *fliC2*), and with ASF6_BFP and ASF6_BRP (for gene *asf6*), respectively. The two amplicons were first cloned separately into the pGEM-T easy vector via TA cloning and then excised via respective restriction enzymes. Both the digested amplicons A and B were cloned in the pSUP202 vector, resulting in pSUP202AB, followed by insertion of a kanamycin resistance gene cassette (derived from pUC4K) between amplicons A and B to disrupt the gene in pSUP202. The final constructs were mobilized into *A. brasilense* Sp7 by conjugation using *E. coli* S17-1 as a donor. Exconjugants were selected on kanamycin-supplemented MMM plates, and the knockout mutants of all three genes were confirmed by PCR amplification using gene-specific primers of *rpoE6, fliC2,* and *asf6* (52).

Non-polar deletion mutants were made by double-homologous recombination using the pK18mobsacB suicide vector. Briefly, about 600 bp upstream and downstream of each deleted gene and a chloramphenicol cassette were amplified individually, then amplified using overlap extension PCR. This single amplified product was then inserted into the pK18mobsacB vector. The resulting plasmid was mobilized via triparental mating into *A. brasilense* and passaged until cells were no longer resistant to the backbone antibiotic kanamycin and could grow on sucrose, indicating that the backbone of the plasmid has been removed from the genome. The resulting mutants are stably chloramphenicol-resistant.

### Complementation and overexpression of the *rpoE6* gene using vector pAK002

For complementation studies and for creating *rpoE6* overexpressing strains, *rpoE6* was PCR amplified with Dream Taq (Fermentas) using primer pair RPOE6OF and RPOE6OR possessing XhoI and XmaI restriction overhangs in their 5′ ends, respectively. The PCR product was digested, purified, and ligated downstream to the constitutive promoter region in a modified pBBR3MCS1 vector (pAK002) (28). The resulting plasmid was conjugatively mobilized into the *rpoE6::km* mutant, and exconjugants were selected on plates supplemented with tetracycline.

### Complementation of *rpoE6, asf6, and fliC2* gene using vector pBBR3MCS1

Complementation for the deletion strains Δ*rpoE6*, Δ*asf6*, and Δ*fliC2* was done by PCR amplification using the following primers: *rpoE6* (*rpoE6* FWD XbaI and *rpoE6* RVS Sac1), *asf6* (*asf6* FWD Xba1 and *asf6* RVS Sac1), and *fliC2* (*fliC2* FWD Xba1 and *fliC2* RVS Sac1). The PCR products were digested, purified, and ligated downstream of the promoter region of pBBR3MCS1. The resulting plasmids were conjugatively mobilized into Δ*rpoE6*, Δ*asf6*, or Δ*fliC2*, respectively, and exconjugants selected on plates supplemented with tetracycline.

### Bacterial growth

To compare the growth pattern of *A. brasilense* Sp7 and its mutants (*rpoE6::km*) along with its complemented strain *rpoE6::km* (pAK002:*rpoE6*) in MMM having 40 mM malate as carbon source (35, 53), LB medium, fructose, and glycerol, ~1 mL of overnight grown cultures of *A. brasilense* Sp7 and all the other strains were pelleted by centrifugation, washed with 0.85% saline, and suspended in MMM to maintain the initial OD_600_ of 0.05. The cultures were grown at 30°C with shaking at 180 rpm for 28 h (in case of MMM and LB medium) and up to 60 h (in case of fructose and glycerol). Growth was monitored every 4 h to plot the growth curves. The bar graphs and growth curves were prepared using the GraphPad Prism software.

### Swimming assay and pellicle formation

For swimming assay, cultures of *A. brasilense* Sp7 and *rpoE6::km* mutant, *fliC2::km* mutant, and complemented strain of *rpoE6::km* mutant, that is, *rpoE6::km* (pAK002:*rpoE6*) were grown overnight. Secondary cultures were inoculated in MM medium and grown to an OD_600_ of ~0.7. Equal-cell-density cultures of all the strains were centrifuged at 4,000 rpm for 3 min. All the supernatants were discarded, and the pellet was washed with 0.85% saline, avoiding loss of any cells. The pellet was then resuspended in 50 µL of MM medium. A 2 µL drop of each strain was placed at the center of MM medium containing 0.25% agar and 8 mM malate as a carbon source. The diameter of the hollow region was captured after 72 h.

For studies related to pellicle forming ability, cultures were grown overnight, secondary cultures were inoculated in MM medium and grown to an OD_600_ of ~ 0.7. Equal-cell-density cultures of all the strains were collected and centrifuged at 4,000 rpm for 3 min, and the pellet was resuspended in 100 µL of nitrogen-free broth (devoid of NH_4_Cl as nitrogen source). An inoculum (50 µL) of each strain was placed at the bottom of the tube having nitrogen-free semi-solid medium with 0.1% agar and 8 mM of malate as a carbon source, and another 50 µL volume of each strain was placed in MMM having NH_4_Cl as a nitrogen source. The tubes were left undisturbed, allowing the formation of a pellicle at the air-liquid interface. The difference in the pellicle-forming ability of each strain was captured after 20 h in both media.

Soft agar swimming assays for *A. brasilense* Sp7 (WT), Δ*rpoE6*, Δ*asf6*, Δ*fliC2,* as well as WT and Δ*rpoE6* carrying an empty vector and Δ*asf6* expressing *asf6* from a plasmid (Δ*asf6+asf6*) were carried out as follows. Cultures were grown overnight, sub-cultured to the log-phase growth, then cells were washed three times in phosphate-buffered saline to get rid of nitrogen from the media. The cells were then normalized to an OD_600_ of 2, and 5 µL of the washed, normalized cultures were each inoculated into the center of a nitrogen-free, 0.2% soft agar (nitrogen-free noble agar) minimal malate media.

### Identification of RpoE6-induced proteins

For the identification of proteins differentially expressed in *A. brasilense* Sp7 due to the overexpression of *rpoE6* by IPTG induction, we employed a previously described method (25). The bacterial culture was grown in MMM. Once the cultures reached an OD_600_ of 1.0 (5 × 10^8^ cells/mL), cells were collected by centrifugation. Total protein extraction was carried out through sonication, followed by centrifugation to remove cell debris. Each protein sample was treated with 5 mM Tris(2-carboxyethyl) phosphine hydrochloride for reduction, then alkylated with 50 mM iodoacetamide. A total of 50 µg of protein lysate was digested with 500 ng of trypsin at 37°C for 14 h. Peptide digests were purified using a C18 silica cartridge to remove salts and other contaminants, followed by drying in a SpeedVac. The resulting dried pellet was resuspended in buffer A (5% acetonitrile and 0.1% formic acid) for further analysis.

All experiments were conducted using the EASY-nLC 1000 system (Thermo Fisher Scientific) in conjunction with an Orbitrap Q-Exactive mass spectrometer (Thermo), equipped with a nano-electrospray ionization source. Peptide mixtures were separated using a 60 cm Viper column packed with C18 resin. Samples were loaded with buffer A and eluted using a 0%–40% gradient of buffer B (95% acetonitrile and 0.1% formic acid) at a flow rate of 250 nL/min over 100 min. Raw data files obtained from mass spectrometry analysis were processed using Proteome Discoverer (v2.2) and searched against the *A. brasilense* Sp7 reference proteome database from UniProt. Carbamidomethylation of cysteine was considered a fixed modification, while methionine oxidation and N-terminal acetylation were treated as variable modifications during database searches. The false discovery rate thresholds for peptide spectrum matching (PSM) and protein identification were both set to 0.01.

### Analysis of co-transcription

To examine the presence of a promoter in the upstream region of *fliC2* and *fliD* genes, co-transcription analysis of *flgL* with *fliC2* and *fliD* was performed. Briefly, total RNA was isolated using the TRIzol method from cells of a late log phase culture of *A. brasilense* Sp7 grown in MMM. RNA isolation and cDNA synthesis were performed as described earlier (54). After treatment with Turbo DNase (Thermo Fisher Scientific, Waltham, USA) for 1 h at 37°C, reverse transcriptase PCR was carried out with 200 ng of RNA and *fliC2-* and *fliD*-specific primers (GS2 and GS1, respectively, Table S1) using a one-step RT-PCR kit (Thermo Fisher Scientific, Waltham, USA). The cycle condition used was 42°C for 60 min followed by heat inactivation at 72°C for 10 min. Positive and negative controls were included in RT-PCR to check primer functionality and DNA contamination. Co-transcription analysis was carried out by specific PCR amplification from cDNA using different sets of primers (Table S1).

### 5′ RACE for mapping the *fliC2* TSS

The TSS of the *fliC2* mRNA was identified using 5′ RACE, following the method described in reference (46). *A. brasilense* Sp7 was grown in MFM to the logarithmic phase, and total RNA was extracted using the TRIzol reagent. After DNase I treatment to remove contaminating genomic DNA, RNA was further purified using the Qiagen RNeasy kit. Subsequently, 5 µg of purified RNA was reverse-transcribed into cDNA using reverse transcriptase and a gene-specific reverse primer (GSP1). The cDNA was enzymatically tailed at its 3′ end with a poly (A) sequence using terminal deoxynucleotidyl transferase, as described in the provider protocol. This polyadenylated cDNA was used as a template for PCR amplification with a high-fidelity DNA polymerase, a second gene-specific primer (GSP2), and an oligo(dT) anchor primer complementary to the poly(A) tail. To enhance specificity, a nested PCR was performed using a third gene-specific primer (GSP3) and the anchor primer. The resulting PCR products were cloned into the pGEM-T Easy vector (Promega), and their sequences were determined by Sanger sequencing.

### Construction of *fliC2::lacZ, fliD::lacZ, rpoE6::lacZ, exaA::lacZ, pqq::lacZ*, *rpoE8::lacZ,* and *fliC2::gusA* transcriptional fusions

Approximately 500 bp regions of DNA located upstream of the start codons of the *fliC2, fliD, rpoE6, exaA, pqq,* and *rpoE8* genes were PCR amplified with their specific primers (Table S1) having XbaI and HindIII restriction sites. After amplification, PCR products were digested with XbaI and HindIII endonucleases and inserted into the similarly digested pCZ750 vector (51) to construct promoter::*lacZ* transcriptional fusions. The confirmed constructs of *fliC2::lacZ, fliD::lacZ, exaA::lacZ,* and *pqq::lacZ* were mobilized into *A. brasilense* and the *rpoE6::km* mutant via conjugation using *E. coli* S17-1 as the donor. Exconjugants were selected on MMM having tetracycline as a selectable marker. For the *fliC2::gusA* transcriptional fusion, a 600 bp region of DNA located upstream of the start codon of *fliC2* was PCR amplified with specific primers (Table S1) with HinDIII and EcoR1 restriction sites. PCR products were then digested with HinDIII and EcoR1 endonucleases and inserted into the digested pFUS1 vector (55) to construct the *fliC2::gusA* transcriptional fusion. This construct was confirmed by PCR and mobilized into *A. brasilense* WT, ∆*rpoE6*, and ∆*asf6* strains by tri-parental conjugation with *E. coli* TOP10 as the donor strain. Exconjugants were selected on tetracycline.

### Two-plasmid assay to verify the dependence of the promoter on a sigma factor

For further studies to find the promoter activated by RpoE6, we used an *E. coli*-based two-plasmid system (56). For this, we transformed *fliC2::lacZ, fliD::lacZ, rpoE6::lacZ,* and *rpoE8::lacZ* fusion plasmids into *E. coli* DH5α individually, and these transformants were then further transformed with plasmids overexpressing *rpoE6* (pMMB206:*rpoE6*). Transformants were selected on plates containing tetracycline and chloramphenicol.

### Quantitation of promoter activity using β-galactosidase and β-glucuronidase

Promoter activity was monitored by measuring the β-galactosidase activity of the *A. brasilense* Sp7 and *rpoE6::km* mutant harboring different constructs (*fliC2::lacZ* and *fliD::lacZ*) in MMM and of *exaA::lacZ* and *pqq::lacZ* in MMM, MFM, and MGM. Briefly, the cultures were grown overnight in LB medium with shaking at 180 rpm and 30°C. Secondary cultures were inoculated in medium with different carbon sources and grown for 8 h at 30°C and 180 rpm to an OD_600_ of ~0.7. Equal-cell-density cultures of all the strains were harvested, washed, and resuspended in a lysis buffer (50 mM phosphate buffer [pH 7.0], 0.1% SDS, 0.27% β-mercaptoethanol, and 100 μL chloroform) for the assay of β-galactosidase activity, which was measured as described earlier (57) by using the formula: 1,000 × (OD_420_ × 1.75 − OD_550_)/time of reaction (in minutes) × volume of culture assayed. To measure β-glucuronidase activity of *fliC2::gusB* constructs in ∆*asf6* and ∆*rpoE6* compared to WT, cultures were grown in minimal medium overnight at 180 rpm and 28°C. Cells were normalized to an OD_600_ of 0.5. Normalized cell cultures were placed in a 96-well plate in triplicate and freeze-thawed three times to break open cells. 4-NPG was used as a substrate for β-glucuronidase, and absorbance was read on a BioTek ELx808IU Absorbance Microplate Reader at OD_405_ every 2 min for an hour. Data were analyzed using Gen5 software. To quantify the data, we used the logarithmic portion of the activity slope and used the formula: (OD_405_(end) − OD_405_(start))/time (in minutes).

### BacTH to examine protein-protein interaction in RpoE6 and Asf6

BacTH system to study *in vivo* protein-protein interaction between RpoE6 and Asf6 was carried out. For this, *rpoE6* and *asf6* genes were PCR amplified (with PKTE6F/PKTE6R and PUTASF6F/PUTASF6R primer pairs, respectively) and cloned into pKT25 and pUT18C, resulting in recombinant plasmids pKT-E6 and pUT-Asf6. Protein-protein interaction was analyzed after the co-transformation and expression of hybrid bait and target proteins from both the recombinant plasmids in the reporter strain BTH101, which was grown in LB medium containing X-gal (40 µg/mL) and IPTG (0.5 mM) with antibiotics ampicillin and kanamycin (56).

### Extracellular protein isolation, SDS-PAGE, and UPLC-MSE analysis

*A. brasilense* was grown overnight in 50 mL of MMAB minimal medium. The cells were gently pelleted (3,000 rpm for 10 min), and the supernatant was removed. The cells were resuspended in 1 mL modified PBS (137 mM NaCl, 2.7 mM KCl, 10 mM Na_2_HPO_4_, 1.8 mM KH_2_PO_4_). The dense cell cultures were then normalized with the modified PBS solution. The cells were then vortexed at max speed for 1 min, pelleted (5,000 RPM for 5 min), and the supernatant was transferred to a new microcentrifuge tube (~1 mL). Pelleting and supernatant transfer were repeated if any cells remained. 500 mL of 2% PEG (~0.70% final concentration) and 3 µL of 0.1 M NaCl (~0.3 mM final concentration) were added to the 1 mL of supernatant that contained the flagella. The solution was incubated on ice for 1 h. The pegylated flagella were then pelleted (2,000 RPM for 5 min), and 80 µL was gently collected from the bottom of the microcentrifuge tube. 20 µL of 6× Laemmli buffer (160 mM Tris [pH 8.0], 4% SDS, 20% Glycerol, 0.01% bromophenol blue, and 40 µM β-mercaptoethanol) was added to isolated flagella and separated on a 12% SDS-PAGE gel at 120V for 1.5 h. The SDS-PAGE gel was then stained with silver nitrate using the Pierce Silver Stain Kit by ThermoFisher Scientific according to the manufacturer’s manual. Briefly, the SDS-PAGE gel was washed in water twice for 5 min, then the proteins were fixed in a 30% ethanol, 10% acetic acid solution for 15 min twice (or overnight). Then, proteins were washed twice for 5 min in 10% ethanol, then twice in MilliQ water. The proteins were then sensitized using Sensitizer Working Solution for 1 min, followed by two brief washes in MilliQ water. Proteins were stained with silver nitrate for 30 min followed by brief washes in MilliQ water. Silver nitrate was then developed with Developer Working Solution for 2–8 min (until bands are visible on gel). Development was stopped with 5% acetic acid.

The Coomassie-stained protein bands corresponding to FliC2 (marked by an arrow in Fig. 8B) were excised from the gel and subjected to in-gel digestion with trypsin. Analyses were conducted using a Waters Synapt XS high-resolution mass spectrometer coupled with a Waters ACQUITY UPLC H-Class system. Chromatographic separation was achieved using a reversed-phase UPLC column with a binary solvent system: mobile phase A consisted of 0.1% formic acid in water, and mobile phase B consisted of 0.1% formic acid in acetonitrile. The system operated in UPLC-MS^E^ mode, enabling simultaneous acquisition of precursor and fragment ion data. Low-energy scans were acquired at a fixed collision energy of 6 eV, while high-energy scans were acquired using a ramped collision energy from 15 to 45 eV in the trap mode. Data were acquired in high-resolution mode to ensure accurate mass determination across the full m/z range. The resulting mass spectra were searched against the FliC2 sequence of *Azospirillum baldaniorum* using the UniProt database.

### PAS-Schiff staining

Extracellular protein isolation and SDS-PAGE were done exactly as with the silver staining procedure described in this paper. Then, the SDS-PAGE gel was stained with the Pierce Glycoprotein Staining Kit by ThermoFisher Scientific according to the manufacturer’s manual.

Briefly, the proteins were fixed in the SDS-PAGE gel in 50% methanol for 30 min, followed by two washes in 3% acetic acid for 10 min. The proteins were then oxidized by the Oxidizing Solution for 15 min, followed by three washes with acetic acid for 5 min each. Proteins were then stained with Glycoprotein Stain for 15 min followed by reduction by a Reducing Solution for 5 min. The gel was then washed extensively with 3% acetic acid followed by a wash with Milli-Q water.

### Mass spectrometry analysis of flagellin proteins from the Δ*asf6* mutant

Two silver-stained gel slices containing the suspected FliC2 bands from the Δ*asf6* mutant were shipped to ITSI Bio for mass spectrometry identification. Briefly, the gel slices were de-stained, alkylated, and trypsin-digested overnight. After digestion, the peptides were extracted and transferred to clean tubes to be dried in a SpeedVac. The samples were then resuspended and Ziptip desalted, dried down, and resuspended for LC-MS/MS analysis. Half of each digested sample was analyzed by nano LC-MS/MS with a Waters M-Class LC system interfaced to a ThermoFisher Exploris 480 mass spectrometer. Peptides were loaded on a trapping column and eluted over a 75 μm analytical column at 350 nL/min. Both columns were packed with XSelect CSH C18 resin (Waters); the trapping column contained a 3.5 μm particle, and the analytical column contained a 2.4 μm particle. The column was heated to 55°C using a column heater (Sonation). The mass spectrometer was operated in data-dependent mode, with the Orbitrap operating at 60,000 FWHM and 15,000 FWHM for MS and MS/MS, respectively. A 1-h gradient was used. The instrument was run with a 3 s cycle for MS and MS/MS. The peptides found were searched against the *A. brasilense* FASTA database downloaded from Uniprot using Proteome Discoverer 2.2 (Thermo Scientific) and the Sequest HT algorithm. Peptides identified with high confidence were considered and used for protein identification. Percolator PSM validator was used for PSM validation in the database searches.

### Transmission electron microscopy

*A. brasilense* cultures were grown overnight and sub-cultured to log growth in MMAB minimal medium. Then, 1 mL of each culture was gently centrifuged (4,000 RPM for 5 min). The supernatant was removed, and samples were gently resuspended in 500 µL medium. All pipetting was done slowly with wide-ended tips to prevent flagellar shearing. Samples were then prepared on carbon-coated TEM disks (CFT200-Cu-50, EMS), by placing 20 µL of the sample on the disk for 1 min, followed by a 15-s rinse with distilled water, followed by negative staining for 1 min using UranyLess EM stain. Samples were then viewed on a JEOL 1400 transmission electron microscope. The width of each flagellum on the TEM micrographs was measured using ImageJ software from a minimum of 30 images for each strain.

### Statistical analysis

Experiments on the measurement of growth were performed in triplicate in three independent experiments. The mean significant values were determined by using the SPSS 17 package software. Analysis of the variance followed by Duncan’s multiple comparison tests (58) was used to analyze the significance between more than two treatments. Differences were considered significant at *P* < 0.05

## SUPPLEMENTAL MATERIAL

Figures S1 to S4; Table S1 jb.00464-25-s0001.pdf Clustal W alignments and primers table.

ASM does not own the copyrights to Supplemental Material that may be linked to, or accessed through, an article. The authors have granted ASM a non-exclusive, world-wide license to publish the Supplemental Material files. Please contact the corresponding author directly for reuse.
