# Supplementary material for: A novel extracytoplasmic function sigma factor (RpoE6) regulates biogenesis of a minor flagellin and other functions in Azospirillum brasilense Sp7
Source: J Bacteriol. 2025 Dec 29;208(1):e00464-25. doi: 10.1128/jb.00464-25 (PMC12826043; doi:10.1128/jb.00464-25)
Supplement: Figures S1 to S4; Table S1 — Clustal W alignments and primers table. [file jb.00464-25-s0001.pdf]

Supplemental Material:

```
Azospirillum          ---------------------------------------------MAAETDEVLMARIRA 15
Magnetospirillum      ------------------------MM------IQRLTTSASSAAALAGLDDETLLARMKD 30
Bradyrhizobium        MSYALDVRAPADGMSSSEPASPEIMTVPWSESVSTVPDGAPPSEDAVFDEDSELLDRLAT 60
Rhodopseudomonas      MTYALDIWAPVEAAPAVMTTPPLAAAMPNEAPCDGAAG------PRPPDDDDRLLARLAG 54
                                                                        *. *: *:

Azospirillum          GDQAAYRALVHRHLKRAYALARRMSGSDAEAEDIAQDAFLQVWQRRDHWTDEGAKFTTWL 75
Magnetospirillum      DDAAAYRLLVERHVDRAYAIALRVLGNVADAEDVAQECLVKVWTHRQSWQDGKAKFSTWL 90
Bradyrhizobium        GDEVAFRLLVERHIDRAYAIALRIVGSAADAEDVVQDTMLKVWTHRGRWQHGRAKFSTWL 120
Rhodopseudomonas      NDEAAFRLLVERHIDRAFSVALRVVGNRADAEDVVQDTMLKVWTHRGQWQQGRAKFSTWL 114
                      .* .*:* **.**:.**:::* *: *. *:***:.*: :::** :*  * .  ***:***

Azospirillum          YRVVLNRCIDHKRRPAGEDLDSVPEPPDHAPDAVTHIQRRQVAARLRDAQDRLPQQQRAA 135
Magnetospirillum      YRVVVNRCIDLRRRPATECLDDVPEPMDDETDSVTRIHRTQVFGRLEQAMGKLPEQQRLA 150
Bradyrhizobium        YRVVSNRCIDLRRKPRTENVDVVPEVPDGKPDAVSVIERAQMSDLLESAMQRLPEQQRVA 180
Rhodopseudomonas      YRVVTNRCIDLRRQPRTDNVDAVPEPADDQPDVVTTIQRDEVSLLLEAAMQRLPEQQRIA 174
                      **** ***** :*:*  : :* ***  *   * *: *.* ::   *. *  :**:*** *

Azospirillum          LALYYNEGLSGAEVATIMQISVTAVESLLKRARQQLRTLLRASAQAARDSFEDG189
Magnetospirillum      LTLSYFDDLGNAEIAEILDTTVSAVESLLKRGRQTLRDRLRRSEHDFRQALAE-203
Bradyrhizobium        VILSYHENMSNGEIAEVMETTVTAVESLLKRGRQQLREMLRRHERDIRGAFTDC234
Rhodopseudomonas      MILSYHQDMSNGEIAEVMETTVAAVESLLKRGRQQLRDLLRRNERDIRHIFTD-227
                      : * * :.:...*:* ::: :*:********.** **  **   :  *  : :
```

Fig. S1: ClustalW alignment of the deduced amino acid sequence of RpoE6 from *Azospirillum brasilense* Sp7 and its orthologs from *Magnetospirillum gryphiswaldense* MSR-1 (MSR1_01160), *Bradyrhizobium japonicum* (BRADO4891), and *Rhodopseudomonas palustris* BisA53 (RPE_0783). The sequences are labeled according to their respective genera: *Azospirillum*, *Magnetospirillum*, *Bradyrhizobium*, and *Rhodopseudomonas*. The consensus line displayed beneath the alignment uses the symbols asterisk (*****), colon (**:**), and period (**.**) to indicate the degree of conservation at each position. An asterisk (*) signifies that all sequences share an identical amino acid at that position, colon (**:**) represents a conserved substitution among amino acids with strongly similar physicochemical properties and period (**.**) indicates a weakly conserved substitution among residues showing more limited similarity.

```
TSH58               ------------------------------------------------------------      0
Az39                ------------------------------------------------------------      0
argentinense        ------------------------------------------------------------      0
Sp7                 ------------------------------------------------------------      0
Sp245               ------------------------------------------------------------      0
humicireducens      ---------------------------------------CCGCCGCGAAGGCGTCCGCAA     21
lipoferum           ----------------------------------------CCGCTGCGAGGGCGCCCGCA     20
thiophilum          ACAAGTACCTGTAAGGGCTCGGGTTTTCTCTTCCGCCGGCAGGGGTGCCGGCCATGCGCG     60
TSA2s               --------------------------------------------TCGCAGGGGCGGCGAA     16
TSH100              -----------------------------------------------CCGGAGGGGCGCA     13

                                             -35 sequence        -10 sequence
TSH58               ---------------GCCGAAGATTCCGCGCCAGCTTTCGCCGCGCCGGCCGTGAGACGC     45
Az39                ---------------GCCAAAGATTCCGCGCCAGCTTTCGCGGCGCCGGCCGTGAGACGC     45
argentinense        ---------------CCGAAAGATTCCGCGCCAGCTTTCGCCGCGCCGGCCGTGAGACGC     45
Sp7                 ---------------TCGAAAGATTCCGCGCCAGCTTTCGCGGCGCAGGCCGTGAGACGC     45
Sp245               ---------------TCGAAAGATTCCGCGCCAGCTTTCGCGGCACCGGCCGTGAGACGC     45
humicireducens      AAAAACTTCTACCCCAATCGAAAAACCCCGCCAGTTTCCGCCGCGTCCCCCGTGAGTCCC     81
lipoferum           AAAAACTTCTACCCCAATCGAAAAACCCCGCCAGTTTCCGCCGCGTCCCCCGTGAGTCCC     80
thiophilum          AAAAAGCGCCAAGCCAACCGAAAAAGCCCGCCAGTTTTCGCCGCGTCCCCCGTGAGTCCC    120
TSA2s               AAAAAGCGCCAACCCGATCGAAAAATCCCGCCAGCTTTCGCCGCTTGCCCCGTGAGTCCC     76
TSH100              AAAAAGCGCTAACCCGATCGAAAAACTGCGCCAGCTTTCGCCCCATCCCCCGTGAGTCCC     73
                                        * *     ****** ** ***  *     ******* * *

                      TSS                                        Shine-Dalgarno (SD)
TSH58               AATGAGGGCTGATGCACAGGCGGCCCCCACCATCCGATCCGGCTCCGACCAGGAGGC-AC    104
Az39                ACTGAGGGCTGATGCACAGGCGGCCCC-ACCATCCGATCAGGCTCCGACCAGGAGGC-AC    103
argentinense        ACTGAGGGCTGATGCACAGGCGGCCCC-ACCATCCGATCAGGCTCCGACCAGGAGGC-AC    103
Sp7                 CATGAGGGCTGATGCACAGGCGGCCCC-ACCATCCGATCAGGCTCCGACCAGGAGGC-AC    103
Sp245               AATGAGGGCTGATGCACAGGCGGCCCC-ACCATCCGATCAGGCTCCGACCAGGAGGC-AC    103
humicireducens      GGTGAGGGTCGCCGAAACGGCCCC-----------------GGTTCGATCAGGAGACCAG    124
lipoferum           GGTGAGGGTCGCCGAAACGGCCCC-----------------GGTTCGATCAGGAGACCAG    123
thiophilum          AGTGAGGGTCGCCGAAACGGCCCC-----------------GGTTCGATCAGGAGACCAG    163
TSA2s               AGTGAGGGTCGCCGAAACGGCCCG-----------------GT-TCGATCAGGAGACCAC    118
TSH100              AGTGAGGGTCGCCGAAACGGCCCG-----------------GT-TCGATCAGGAGACCAC    115
                      ******  *  * *  ***                    *   *** ****** * *

TSH58               ATC 107
Az39                ATC 106
argentinense        ATC 106
Sp7                 ATC 106
Sp245               ATC 106
humicireducens      ATC 127
lipoferum           ATC 126
thiophilum          ATC 166
TSA2s               ATC 121
TSH100              ATC 118
                    ***
```

Fig. S2: Clustal Omega alignment of the non-coding upstream region of *fliC* from various *Azospirillum* species. The guanine (G) highlighted in yellow indicates the transcription start site (TSS) of *fliC* in *Azospirillum brasilense* Sp7, as determined by 5′ RACE. The Shine-Dalgarno (SD) sequence is highlighted in green, the predicted −10 promoter element in blue, and the predicted −35 promoter element in red. These regulatory motifs were inferred based on sequence conservation among the species and their relative positions to the TSS. Strain abbreviations: **TSH58**, *Azospirillum* sp. TSH58; **Az39**, *A. brasilense* Az39; **argentinense**, *A. argentinense*; **Sp7**, *A. brasilense* Sp7; **Sp245**, *A. baldaniorum* Sp245; **humicireducens**, *A. humicireducens*; **lipoferum**, *A. lipoferum*; **thiophilum**, *A. thiophilum*; **TSA2s**, *Azospirillum* sp. TSA2s; **TSH100**, *Azospirillum* sp. TSH100.

```
R.palustris       MPAISTNTAANSAVRYLNINSAQETSSLSKLASGSRITSASDDAAGLAISTRISSDVTTL    60
B.japonicum       MPAISTNVAANSAVRYLNINSSQETSSLSKLSSGSRITSASDDAAGLAISTRISSDITTL    60
A.brasilense      MPVISTNTAANSALRYLNINSENQSSSVSKIASGSRITKASDDAAGLAVGTSLTSDITVL    60
A.lipoferum       MPAITTNTASNSALRYLNINSENQSDSVSKIASGSRITKASDDAAGLAVGTSLQSDITVL    60
                  **.*:**.*:***:******* :::.*:**::******.*********:.* : **:*.*

R.palustris       QQAATNASQAVAILQTADGGASNISDILARMKSLASESASGTTTD-SSRAYINSEFSQLS    119
B.japonicum       QQAATNASQATSILQTADGGASNISDILARMKSLASESASGTTVDSSSRSYIQSEFSQLI    120
A.brasilense      KQAATNASHGSSILQAADGGMSRVSDIVQRMRSLATQSLSGAVTD-TERGYLDAEFQQLI    119
A.lipoferum       NQAATNASHGSSILQTADGGMSSISDIVQRMRSLATQSLSGSVTD-TERDYLDAEFQQLQ    119
                  :*******:. :***:**** * :***: **:***::* **:..* :.* *:::**.**

R.palustris       SQIDSIASGTRYSSQSLLDGTSVFASGVAVLVGTQSSDSITITLSNLKASTLGVSTLDVS    179
B.japonicum       SEIDSIASGTRYSGTSLLDGTSSFSTGVNVLVGSNSTDVIQIKLSSLTSTALGVSTLDVS    180
A.brasilense      EEIDGIASGTRFNDDPLLDGNGAWASGVDFRVGTESTDKITVTIANVNTTGLSIGTLDVG    179
A.lipoferum       DEIDGIASGTRFNDESLLDGTEQWASGVGFRVGTTATDSITVTIDDVTTTGLGINTLDVG    179
                  .:**.******:..  ****.  :::** . **: ::* * :.: .:.:: *.:.****.

R.palustris       SLSGATTALSALDTAINTVSSARASIGAQESRFNFSADSISTQTQNLQSANSAIKDVDIA    239
B.japonicum       TQTGASTALTTLDTAISNVSAARADIGAQESRFNFSADSISTQTQNLQSANSAIKDVDIA    240
A.brasilense      TSATASAALTALDTAVETLSSARADVGALISRFEFRGQVLDTSIENTEAAQSAIMDVDVA    239
A.lipoferum       TSATAAAALTALDAAVTTLSDARADVGALISRFEFRSDMISTTIENTEAAQSAIMDVDVA    239
                  : : *::**::**:*: .:* ***.:**  ***:* .: :.*  :* ::*:*** ***:*

R.palustris       AEQAKLSSAEVKTQAAVSAEAAANQMPQYLLKLLG    274
B.japonicum       SEQAKLSSAQVKTQAAVSAEAAANQIPQYLLKLLG    275
A.brasilense      AEQAELASTKVLTQAAIAVLSQANEMPQNLLQLLR    274
A.lipoferum       AQQSQLASEKVLTQAAIAVLSQANSMPENLLSLLR    274
                  ::*::*:* :* ****::. : **.:*: **.**
```

Fig. S3. ClustalW alignment of the deduced amino acid sequence of FliC2 from *Azospirillum brasilense* Sp7 and its orthologs in *Rhodopseudomonas palustris* BisA53 (RPE_0782), *Bradyrhizobium japonicum* (BRADO4890), and *Azospirillum lipoferum* 4B. The sequences are labeled *R. palustris*, *B. japonicum*, *A. brasilense*, and *A. lipoferum*, respectively. The consensus line displayed beneath the alignment uses the symbols asterisk (*), colon (:), and period (.) to indicate the degree of conservation at each position. An asterisk (*) signifies that all sequences share an identical amino acid at that position, colon (:) represents a conserved substitution among amino acids with strongly similar physicochemical properties and period (.) indicates a weakly conserved substitution among residues showing more limited similarity.

```
FliC1   MATSDISLSSSMRTNLLQLQGVQDSIAKKQNILSTGNKINSALDGPTSFFAAKNLTQRAG
FliC2   --MPVISTNTAANSALRYLNINSENQSSSVSKIASGSRITKASDDAAGLAVGTSLTSDIT
            . ** .:: .: *  *:  .:. :.. . :::*.:*..* *..:.: ....**.

FliC1   DLTALKDTMGQAISTIKAADKGLSSIDDMIEQARGLTTAAYSALGTDAGSVATRKSLSNQ
FliC2   VLKQAATNASHGSSILQAADGGMSRVSDIVQRMRSLATQSLSGAVTDT----ERGYLDAE
         *.     . .:. * ::*** *:* :.*:::: *.*:* : *.  **:     *  *. :

FliC1   FNALKEQIDKLAGDSAYGGKNLLAGNGLRLDSTSDSRRAVNTIQGIENARITNVVSTDTY
FliC2   FQQLIEEIDGIASGTRFNDDPLLDGNGAWASG----------------------------
        *: * *:** :*..: :... ** ***    ..

FliC1   TVRVKGDGSIEGAAGEINSAEMAHGLVGLKLSGTMTTSAGSFSDVQIEVRGASGRERSFI
FliC2   ------------------------------------------------------------

FliC1   VTDGNESRTITYFDNSQTIAANTTRTASTGTAQLTNVTLGGTIEAGDSFTITVEDQTFTY
FliC2   ------------------------------------------------------------

FliC1   TATAGDVATGQTARNNIANQLKASINNALGANGRLSGKDVQTVTVNSGGTITLSGATTTN
FliC2   ------------------------------------------------------------

FliC1   SAREMTVKATAENALTKRISESFASGTIVSFTVDRKLLEQAANGGNGVSSIEKKVDIQIS
FliC2   ------------------------------------------------------------

FliC1   VSNLSGATVTRDGMSKRGEGKLADGENAYAFDTGTVRFNVDQKTIKQAAAANSAANLVSV
FliC2   ------------------------------------------------------------

FliC1   QVTDANTSNDLTVQLNERNTNAITVKAQNLTTSGQGLRLDYAQNDWTDRADIDKAVASID
FliC2   ----------VDFRVGTESTDKITVTIANVNTTG----LSIGTLDVGTSATASAALTALD
                  : .::. ..*: ***.  *:.*:*    *. .  *    *  . *::::*

FliC1   YAKQNLRSSSQTLSTNLNVITTRENFTKEFSDVLTEGASKLTLADQNEEGANLIMLQTRQ
FliC2   TAVETLSSARADVGALISRFEFRGQVLDTSIENTEAAQSAIMDVDVAAEQAELASTKVLT
         * :.* *:   :.: :. :  * :. .   :    . * :  .*   * *:*   :.

FliC1   QLGTIALSLANQSQQSILRLF-
FliC2   QAAIAVLSQANEMPQNLLQLLR
        * .  .** **:  *.:*:*:
```

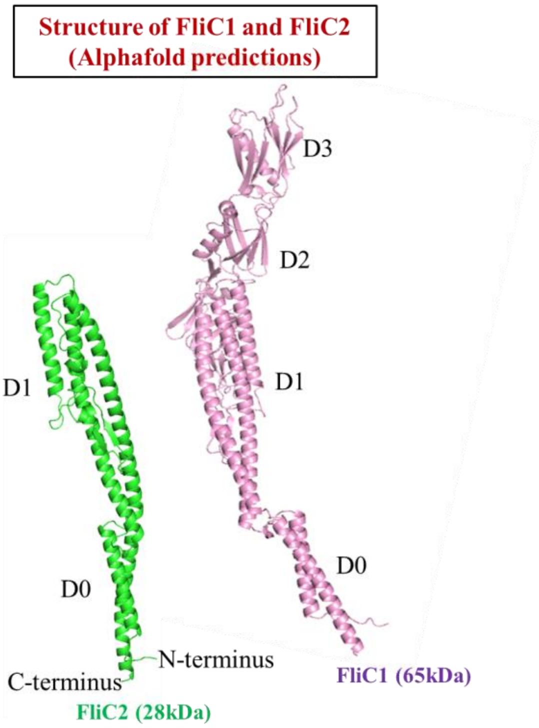

Fig. S4. ClustalW alignment and structural domain comparison of the deduced amino acid sequences of FliC1 and FliC2 in *Azospirillum brasilense* Sp7. The alignment highlights the absence of the D2 and D3 domains in FliC2 compared to FliC1. The alignment highlights the absence of the D2 and D3 domains in FliC2 compared to FliC1.

TABLE S1. Primers used in this study

**Primers**	**Sequence 5----3' direction**
RPOE6AF	CGGAATTCCGACAACACCGACATGAC
RPOE6AR	GAAGATCTCAGCACCACCCGATAGAG
RPOE6BF	GAAGATCTAACCGCTGCATTGACCAC
RPOE6BR	AACTGCAGCTGACCACCAACCTGGATC
FLIC2AF	AACTGCAGCGTGAACGTCAACGAGGAAAC
FLIC2AR	GAAGATCTGCTGGCGATCTTGGAGACCG
FLIC2BF	GAAGATCTCAAGGTGCTGACCCAGGCG
FLIC2BR	CGGAATTCGTGAGGATCAGCTGGTACTG
ASF6AF	GGAATTCTCTGGACTCGGTGCCCGAG
ASF6AR	GAAGATCTGCGTCGCGCGGGGCCAGC
ASF6BF	GAAGATCTGCCTGACGCCGCTCCTTTGC
ASF6BR	AACTGCAGCGCTGACCGCCAATCTGGATC
RPOE6OF	CCGCTCGAGGATGCGCGCGGCGGCCTGTTC
RPOE6OR	TCCCCCCGGGTCATCCGTCTTCGAACGAATC
RPOE6PF	CGGGATCCGGCTTGGCGGCGGAGACC
RPOE6PR	CCCAAGCTTCGCGTCATCCGTCTTCGAAC
RPOE8PF	CGGGATCCGTGGCGGTGATCGACGAGG
RPOE8PR	CCCAAGCTTTCATTTCTCGTCGCCGTCCTTC
ZFLIC2F	GCTCTAGAGATGTGCCTCCTGGTCGGA
ZFLIC2R	CCCAAGCTTCGAACCATCGACCGCCATCTG
ZFLIDF	GCTCTAGAGCGGGTCTCCGGGGGCGG
ZFLIDR	CCCAAGCTTGGCTGTCTACCCACTCCTTCC
ZRPOE6F	CCCAAGCTTGAGATCCATGGATCATGCGC
ZRPOE6R	GCTCTAGAGCCGGTCATGCTCCTGCC
ZRPOE8F	CCCAAGCTTGACGGTCCGTGTGTCTGGCC
ZRPOE8R	GCTCTAGAACGGCCATCCTCTGCTCCTTC
PKTE6F	GCTCTAGAGGCTTGGCGGCGGAGACC
PKTE6R	CGGAATTCCGCGTCATCCGTCTTCGAAC
PUTHPF	GCTCTAGACGATGACCGAAGACGAGTTTC
PUTHPR	CGGAATTCCACGAGCCGCCCGGTCTGA
GS1	ACAGATAGGCGGTGC
GS2	CTTGTCCGTGCTTTCC
FLIDR	CGGTCGAGGATGAGGTTGAG
FLIC2F	GTCGGCGATCATGGACGTGG
FLIC2R	CGCCGCCGTGTTGGTGGAG
FLGLF	GGCGGCGGACGGGCTGAC
GSP1	TTTCCGTGCCCACGC
GSP2	GGAAATCCACGCCGC
GSP3	CGGGTCGTCGTTGAAGCGGG

Oligo-dT anchor primer	GACCACGCGTATCGATGTCGACTTTTTTTTTTTTTTTTTTTTV
Anchor primer	GACCACGCGTATCGATGTCGAC
p*fliC2* FWD HINDIII	ATAGCGAAGCTTTATTTCCAGGCCGCCGACGATTTCAT
p*fliC2* RVS EcoRI	ATACGCGAATTCGCCTCCTGGTCGGAGCCTGAT
Asf6 FWD XbaI	ataTCTAGAATGACCGAAGACGAGTTTCGCGAGC
Asf6 RVS SacI	ataGAGCTCCACGCTTTCTGAAGAGGAAGGGATCAGACC
RpoE6 FWD XbaI	ataTCTAGAACGTCCGTCCGAAACCCTTTCCAAA
RpoE6 RVS SacI	ataGAGCTCCGCGTCATCCGTCTTCGAACGAATC
FliC2 FWD XbaI	ataTCTAGAATGCCCGTCATCTCCACCAACAC
FliC2 RVS SacI	ataGAGCTCGGGTAGACAGCCTTACCGCAGCAG
Asf6 UP FWD XbaI	gaagcagcTCTAGActacaACACGTCCGTCCGAAACCCTTTCCA
Asf6 UP RVS	tgactagttcagttagctaCATCGCGTCATCCGTCTTCGAACGAAT
Asf6 DOWN FWD	gctagctgagtaggtagGTCTGATCCCTTCCTCTTCAGAAAGCGTGAATG
Asf6 DOWN RVS HINDIII	gtcgAAGCTTccccggaatCGTGGCGCAGCCAGTTGATGAC
Asf6 Mutation Check RVS	GCCGGTCAGGTGGTCATCGAGTG
RpoE6 UP FWD XbaI	gaagcagcTCTAGActacaCCCGCATCCCCATCCGGAGAAG
RpoE6 UP RVS	tgactagttcagttagctaCTGCCGGACACGGATTTGGAAAGG
RpoE6 DOWN FWD	gctagctgagtaggtagATGACCGAAGACGAGTTTCGCGAGC
RpoE6 DOWN RVS HINDIII	gtcgAAGCTTccccggaatGGGACTGGACCCATCTCGGCAG
RpoE6 Mutation Check RVS	CGTGGCGCAGCCAGTTGATGAC
FliC2 UP FWD XbaI	gaagcagcTCTAGActacaTATTTCCAGGCCGCCGACGATTTCAT
FliC2 UP RVS	tgactagttcagttagctaTCCTGGTCGGAGCCTGATCGGA
FliC2 DOWN FWD	gctagctgagtaggtagGGCTGTCTACCCACTCCTTCCCTCT
FliC2 DOWN RVS HINDIII	gtcgAAGCTTccccggaatGGATCAGCTGGTACTGGCTGTCCGTCA
FliC2 Mutation Check RVS	CAGCGCCGTCTCGTCCACTTT
CmR insert R	CTACCTACTCAGCTAGCTTACGCCCCGCCCTGCCACTC
CmR insert F v. 2	TAGCTAACTGAACTAGTCACTCTTGAAATAAGATCACTACCGGGCG
